# A Gene Optimization Strategy that Enhances Production of Fully Functional P-Glycoprotein in *Pichia pastoris*

**DOI:** 10.1371/journal.pone.0022577

**Published:** 2011-08-03

**Authors:** Jiangping Bai, Douglas J. Swartz, Irina I. Protasevich, Christie G. Brouillette, Patina M. Harrell, Ellen Hildebrandt, Brigitte Gasser, Diethard Mattanovich, Andrew Ward, Geoffrey Chang, Ina L. Urbatsch

**Affiliations:** 1 Department of Cell Biology and Biochemistry, and Center for Membrane Protein Research, Texas Tech University Health Sciences Center, Lubbock, Texas, United States of America; 2 Department of Chemistry, University of Alabama at Birmingham, Birmingham, Alabama, United States of America; 3 Center for Biophysical Sciences and Engineering, University of Alabama at Birmingham, Birmingham, Alabama, United States of America; 4 Department of Biotechnology, University of Natural Resources and Life Sciences, Vienna, Austria; 5 Department of Molecular Biology, The Scripps Research Institute, La Jolla, California, United States of America; University of Cambridge, United Kingdom

## Abstract

**Background:**

Structural and biochemical studies of mammalian membrane proteins remain hampered by inefficient production of pure protein. We explored codon optimization based on highly expressed *Pichia pastoris* genes to enhance co-translational folding and production of P-glycoprotein (Pgp), an ATP-dependent drug efflux pump involved in multidrug resistance of cancers.

**Methodology/Principal Findings:**

Codon-optimized “Opti-Pgp” and wild-type Pgp, identical in primary protein sequence, were rigorously analyzed for differences in function or solution structure. Yeast expression levels and yield of purified protein from *P. pastoris* (∼130 mg per kg cells) were about three-fold higher for Opti-Pgp than for wild-type protein. Opti-Pgp conveyed full *in vivo* drug resistance against multiple anticancer and fungicidal drugs. ATP hydrolysis by purified Opti-Pgp was strongly stimulated ∼15-fold by verapamil and inhibited by cyclosporine A with binding constants of 4.2±2.2 µM and 1.1±0.26 µM, indistinguishable from wild-type Pgp. Maximum turnover number was 2.1±0.28 µmol/min/mg and was enhanced by 1.2-fold over wild-type Pgp, likely due to higher purity of Opti-Pgp preparations. Analysis of purified wild-type and Opti-Pgp by CD, DSC and limited proteolysis suggested similar secondary and ternary structure. Addition of lipid increased the thermal stability from T_m_ ∼40°C to 49°C, and the total unfolding enthalpy. The increase in folded state may account for the increase in drug-stimulated ATPase activity seen in presence of lipids.

**Conclusion:**

The significantly higher yields of protein in the native folded state, higher purity and improved function establish the value of our gene optimization approach, and provide a basis to improve production of other membrane proteins.

## Introduction

P-glycoprotein (Pgp^2^, also known as multidrug resistance protein MDR1 or ABCB1) is a plasma membrane protein that has the ability to pump a wide range of hydrophobic compounds out of cells. It has particular relevance to chemotherapy, because it is able to prevent accumulation of many anti-cancer drugs in cells, thus conferring multidrug resistance (MDR) [1]. Therefore, Pgp has been a target for improving cancer treatment since its discovery more than three decades ago [2], [3], [4]. Pgp has also been therapeutic targeted for its role in MDR of HIV, epilepsy, and psychiatric illnesses [5], [6], [7], [8]. Pgp is an ABC transporter that requires the energy from ATP binding and hydrolysis in the nucleotide binding domains (NBDs) to drive drug transport across the membrane. Drug binding to the transmembrane domains (TMDs) typically stimulates ATP hydrolysis in the NBDs [9], while inhibitors may compete with drug binding at the polyspecific drug binding sites and so block transport activity and/or ATP hydrolysis. Pgp, like other ABC transporters, is thought to alternate between an inward-facing, drug-binding competent conformation with the transmembrane domains (TMDs) open to the cytoplasm, and an outward-facing, drug-releasing conformation with the TMDs accessible to the extracellular space [10]. We recently solved an X-ray structure of this mammalian ABC transporter in the inward-facing conformation at 3.8 Å resolution [11]. Co-crystal structures with two inhibitors provided a first glimpse of the interactions between bound inhibitors and the drug binding site residues. However, much work remains to fully understand the interaction of Pgp with drugs and inhibitors and the molecular mechanism of drug export. For these endeavors, large-scale production of the fully functional protein is essential.

Previously, we expressed Pgp in the yeast *Pichia pastoris* and purified the protein in its fully active form [12], [13]. This yeast grows to very high densities in fermentor cultures providing ample source material. However, the modest expression level of this integral membrane protein still presents a bottleneck to large scale protein production. Analysis of genes highly expressed in the yeast *Saccharomyces cerevisiae* has revealed a strong relationship between tRNA multiplicity and codon selection [14], [15], [16], suggesting that codon usage bias may be one of the factors that lead to inefficient translation and limit protein production. While effective *E. coli* strains have been developed to overcome the codon bias problem in that expression platform [17], relatively little has been done to address the problem in *P. pastoris* [18], [19], [20], [21], [22]. Previous gene optimization procedures were commonly based on the Kazusa codon usage database (http://www.kazusa.or.jp/codon/), but an important limitation is that it does not discriminate between poorly and highly expressed genes. Because translation efficiency of more highly expressed genes may be especially sensitive to codon usage, attention to this aspect of gene sequence may be profitable for maximizing protein expression.

In this study, we generated a codon usage table specific for highly expressed genes in *P. pastoris* and found that codon usage bias for this subgroup is significantly more stringent than the average codon usage of genes present in the Kazusa database and in the recently published *P. pastoris* genome [23], [24]. We then codon-adjusted the sequence of the Pgp-encoding *mdr3* gene, taking into account relative codon frequencies for each amino acid, as well as optimizing GC content and controlling for mRNA instabilities. We demonstrate that expression of Pgp was significantly increased using this strategy. Previous studies found that silent single nucleotide polymorphisms can alter Pgp function and tertiary structure; therefore it was imperative to ascertain that Opti-Pgp retained its functionality, polyspecific drug interactions and folded state. Opti-Pgp was fully active *in vivo* in yeast drug resistance and mating assays. Furthermore, the quality of the purified protein was improved as judged by size-exclusion chromatography and by ATP hydrolysis rates. Consistent with its activity, the codon-optimized protein exhibited secondary and tertiary structure similar to wild-type (WT) Pgp based on circular dichroic spectroscopy and differential scanning calorimetry analysis of its thermal unfolding properties, respectively.

## Materials and Methods

### Materials

n-Dodecyl-β-D-maltopyranoside (DDM) was obtained from Inalco Pharmaceutical (Milan, Italy), and *E. coli* polar lipid extract from Avanti Polar Lipids (Alabaster, AL). Doxorubicin and trypsin were from Sigma-Aldrich (St. Louis, MO). FK506 and valinomycin were from AG Scientific (San Diego, CA).

### Optimization of the Pgp gene

**Figure 1:**
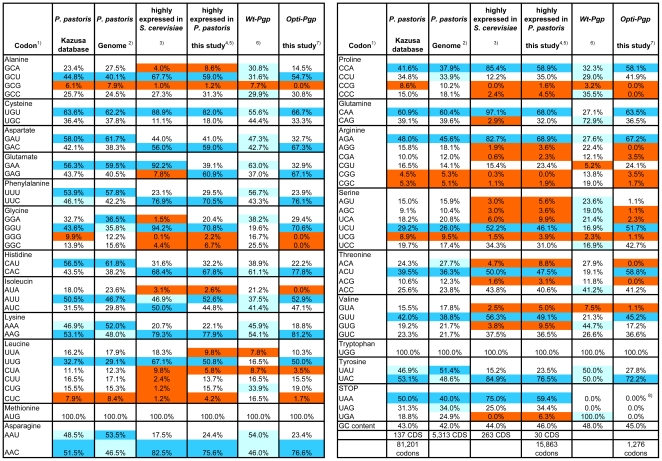
Comparison of codon usage. ^1)^ Codons with low frequency (<10%) are highlighted in red. The most preferred codon for each amino acid is highlighted in dark blue. Most frequent codons (and second most frequent, if within 10% of the first) in WT-Pgp are highlighted in light blue. ^2)^ From [23]. Five codons occur at low frequencies in the Kazusa and Genome databases, which do not discriminate between poorly and high expressed genes, e.g. the codons for Ala (GCG), Leu (CUC), Arg (CGG and CGC) and Ser (UGG). Some preferred codons differ between the Kazusa and the *Pichia* genome databases, namely the codons for Gly, Lys and Asn; this is likely due to the limited number of 137 CDS's represented in the former. ^3)^ From [15]. ^4)^ The codon usage analysis was updated March 2011 to include the 30 most highly expressed genes in *P. pastoris* (see Table S1) based on proteome analysis [26], [27], [28]. Incidentally, all 30 genes are also among the 100 most highly transcribed genes seen in microarrays (Mattanovich, unpublished observations). ^5)^ In highly expressed genes, an additional 18 codons occur at low frequencies, e.g. the codons for Ala (GCA), Gly (GGG and GGC), Ile (AUA), Leu (CUA, CUC and UUA), Pro CCG and CCC), Arg (AGG and CGA), Ser (AGU, AGC and UCA), Thr (ACA and ACG) and Val (GUA and GUG). Comparison of the preferred codon between highly expressed *Pichia* genes and the Kazusa/genome databases revealed an inverted preference for the Asp codon AAC over AAU, CAC over CAU for His and UUC over UUU for Phe. There was also a strong preference for the Lys codon AAG over AAA, AAC over AAU for Asn, and UAC over UAU for Tyr among highly expressed *Pichia* genes. Notably, the codon choice for Glu differed between highly expressed genes of the two yeasts with *S. cerevisiae* showing a clear preference for GAA (92%) whereas *P. pastoris* has a more balanced distribution of 61:39% between GAA and GAG. ^6)^ The native Pgp revealed extensive codon bias, with pronounced over-representation of codons occurring at low frequency among highly expressed *Pichia* genes; viz. codons used for Ala (GCG), Gly (GGG, and GGC), Ile (AUA), Leu (CUA and CUC), Pro (CCC and CCG), Arg (AGG, CGA, CGG and CGC), Ser (AGC, AGU, UCA and UCG), Thr (ACG), and Val (GUA). The native gene also under-represented the *Pichia* higher frequency codons including the preferred codons (compare dark and light blue in columns 4 and 5). For example, the three codons for Ala (GCA, GCU and GCC) are used at about equal frequencies (30–32%) in WT-Pgp whereas highly expressed *Pichia* genes show a clear preference for GCU (59%) over GCC (31%) and GCA (9%). ^7)^ For gene optimization all low-frequency codons (<8%) were set to zero and the distribution of frequencies adjusted to those of highly expressed *Pichia* genes. In some cases, desirable restriction enzyme sites required the presence of a low-frequency codon. ^8)^ The C-terminal His_6_-tag and STOP codons were provided by the pLIC-H_6_ vector (Fig. S2) and were CAT CAT CAT CAT CAT CAT TGA.

The mouse *mdr3* nucleotide sequence (accession number NM_011076), with all three N-glycosylation sites N83, N87 and N90 replaced by glutamine [25] was optimized. Codon substitutions were based on a usage frequency table we calculated for 30 native genes (15,863 codons) known to be highly expressed in *P. pastoris*. These include ACO1 (Pas_chr1-3_0104), ACS1 (Pas_chr2-1_0767), AOX1 (Pas_chr4_0821, PPU96967); CAT2 (Pas_chr3_0069), CCP1 (Pas_chr2-2_0127), CDC19 (Pas_chr2-1_0769), CTA1 (Pas_chr2-2_0131), ENO1 (Pas_chr3_0082), FBA1 (Pas_chr1-1_0072), FDH1 (Pas_chr3_0932), FLD1 (AF066054), GDH3 (Pas_chr1-1_0107), GPM1 (Pas_chr3_0826), GUT2 (Pas_chr3_0579), HSP82 (Pas_chr1-4_0130), ICL1 (Pas_chr1-4_0338), ILV5 (Pas_chr1-1_0432), KAR2 (Pas_chr2-1_0140, AY965684), MDH1 (Pas_chr2-1_0238), MET6 (Pas_chr2-1_0160, AY601648), PDI1 (Pas_chr4_0844, AJ302014), PGK1 (Pas_chr1-4_0292), PIL1 (Pas_chr1-4_0569), RPP0 (Pas_chr1-3_0068), SSA3 (Pas_chr3_0230), SSB2 (Pas_chr3_0731), SSC1 (Pas_chr3_0365), TDH3 (Pas_chr2-1_0437, also called GAP, PPU62648), TEF2 (Pas_FragB_0052, AY219033), YEF3 (Pas_chr4_0038, also called TEF3, AB018536) ([26], [27], [28], [29], [30] and Mattanovich, unpublished results). Codon usage frequency of the collective open reading frames was calculated using the Entelechon software (http://www.entelechon.com/2008/10/codon-usage-table-analysis/). For gene optimization, the software Leto was used (version 1.0.11, Entelechon, Germany), imposing the codon usage for the 30 highly expressed genes (see Fig. 1) except in cases where codons were retained in order to preserve desirable restriction enzyme sites. Furthermore, extended secondary mRNA structure, long range repeats including AT-rich and GC-rich regions and cryptic splice sites were removed and the GC content adjusted to 45%. The Leto software identifies inverted repeats (hairpin stems) with ≤10% mismatches with a distance between inverted repeats (hairpin loops) of at least four nucleotides. For identification of cryptic splice acceptor and donor sites, a hidden Markov model is built in using confirmed splice sites in *S. cerevisiae* gene sequences retrieved from NCBI Entrez. The software is a multi-objective gene algorithm and takes into account all these parameters at all times to simultaneously optimize over the entire sequence of the gene. Unique restriction sites were introduced (Fig. S1A) to facilitate later genetic manipulations. The optimized “*opti-mdr3”* gene was synthesized by GeneArt (Regensburg, Germany).

### Cloning of Opti-Pgp and Expression in S. cerevisiae

The full-length coding sequence of *opti-mdr3* was first cloned into the *P. pastoris* vector pLIC-H_6_ via ligation-independent cloning as described in [31], introducing a Kozak-like sequence around the ATG start codon and a His_6_-tag at the C-terminus (Fig. S2). For direct comparison of gene expression, WT *mdr3* was also cloned into pLIC-H_6_ using the same strategy (simultaneously removing 5′- and 3′-untranslated regions). The resulting plasmids were named pLIC-*opti-mdr3-*H_6_ and pLIC-*mdr3-*H_6_. Then, *opti-mdr3* (including flanking *BstBI* and *AgeI* restriction sites) was PCR amplified using PfuUltra II (Stratagene) and primers 5′-TTCGAAAAAAAAATGGAGTTGG-3′ (forward) and 5′-ACCGGTTCAATGGTGGTGATGGTGGTGCTCGAGAGATCTTTTGGC-3′ (reverse), then cloned into the PvuII and BamHI sites (blunt-ended with T4-DNA polymerase) of the pVT vector [12], [32] to generate pVT-*opti-mdr3*. The integrated full-length ORFs from three individual plasmids were confirmed by DNA sequencing. These three plasmids as well as the pVT vector control and the WT gene in pVT (previously named pVT-*mdr3.5* [*12*]), were transformed into *S. cerevisiae* strain JPY201 (*MAT* **a** *ste6*Δ*ura3*) and selected on uracil-deficient medium as described [12]. 50 to 100 colonies of each transformant were collected into 5 ml of uracil-deficient medium and the mass populations stored at 4°C for up to two weeks; aliquots were frozen as glycerol stocks at −70°C. Mass populations were grown overnight in uracil deficient medium to an OD_600_ of 1 for protein expression and functional analyses. For Western blot analysis, microsomal membranes were processed from 10 ml cultures [13] and the protein concentrations determined with the Bradford protein assay (BioRad) using BSA as a standard. Equal amounts of membrane protein (15 µg) were resolved on SDS-gels, transferred to a nitrocellulose membrane and stained with Ponceau S (total protein loading control). After washing, the immunoblots were developed with the monoclonal C219 antibody (Covance SIG-38710) and the enhanced chemiluminescence SuperSignal West Pico ECL kit (Pierce). The films from different exposure times were scanned and analyzed using the NIH software package ImageJ (http://rsbweb.nih.gov/ij/).

### Functional analysis of Opti-Pgp in S. cerevisiae

FK506 resistance and mating assays were as previously described [12] with the following modifications. To measure FK506-resistant growth, overnight cultures were grown in uracil-deficient medium, diluted to an OD_600_ of 0.05, seeded into sterile 96 well plates in triplicate and grown in YPD medium at 30°C in the absence or presence of FK506, valinomycin [12], [33], or doxorubicin. OD_600_ was measured at 2 hour intervals for 30 hours in a microplate reader (Benchmark Plus, BioRad) after vigorous mixing. Drugs were dissolved in dimethylsulfoxide and diluted into the plate medium such that the final concentration of solvent was ≤1%.

For mating assays, mass populations were diluted to OD_600_ of 0.6, and 0.75 ml were spotted with 0.25 ml of α-type tester strain DC17 (OD_600_ of 1.2) onto a 22 mm 0.45 µm HA filter (Millipore, cat no SA1J791H5), placed on a YPD plate and incubated for 4 hours, then plated in duplicate on minimal and uracil-deficient medium as described [12], [34]. Mating frequency was calculated as the ratio of transformed cells forming diploid colonies on selective medium to the total number of cells introduced in the assay. Statistical analysis of the functional assays was done with the SigmaPlot 11 software using One Way ANOVA with the pairwise multiple comparison Tukey test.

### Expression and purification of WT- and Opti-Pgp from P. pastoris

Transformation of *P. pastoris* strain KM71H and expression analysis were as previously described [31], [35]. Selected strains were grown in a BioFlow IV fermentor and the proteins purified as previously described [13] with the following modifications: 10 mM DTT was included during cell breakage in a glass bead beater to fully reduce the proteins, and all buffers for membrane preparation and chromatography were supplemented with 1 mM β-mercaptoethanol and 0.1 mM tris(2-carboxyethyl)phosphine (TCEP) to keep proteins reduced. Proteins were concentrated to approximately 1 mg/ml using YM-100 Ultrafilters (Millipore). The concentrated protein was aliquoted and stored at −80°C. For gel filtration chromatography, protein was concentrated to 4 mg/ml and 0.5 ml chromatographed on Superose 6B (10×300 mm, GE Healthcare) in 20 mM Hepes-NaOH pH 7.4, 10% glycerol, 50 mM NaCl, 1 mM DTT and 0.2% n-Dodecyl-β-D-maltopyranoside (DDM) using an Äkta Purifier chromatography system (GE Healthcare). Pgp concentrations were routinely determined by UV spectroscopy at 280 nm using a calculated extinction coefficient of 1.28 per mg/ml. Serial dilutions of WT- and Opti-Pgp preparations were further assayed side-by-side with the colorimetric BCA protein assay (Pierce) using BSA with appropriate buffer controls as a standard; the two assays gave essentially the same results. Finally, increasing concentrations of different protein preparations were resolved side-by-side on Coomassie-stained SDS-gels, individual lanes were scanned and the amount of protein in the Pgp and other protein bands quantitated using ImageJ (http://rsbweb.nih.gov/ij/). The latter method permits visual inspection as well as quantitative validation of samples and allows for direct comparison of the Pgp content of the samples.

### ATPase assays

Purified Pgp in 0.1% DDM was mixed with 10 mM DTT on ice for 5 min, then activated with 1% *E. coli* polar lipids for 15 min at room temperature followed by 30 s bath sonication as described [13]. ATPase activity was measured at 37°C in a coupled assay utilizing an ATP-regenerating system [36]. For each well of a 96-well plate, 10 µl (5 µg) of activated wild type (WT) Pgp or Opti-Pgp was added to 200 µl of assay medium containing 10 mM ATP, 12 mM MgSO_4_, 3 mM phosphoenolpyruvate, 0.3 mM NADH, 0.5 mg/ml of lactate dehydrogenase, 0.5 mg/ml of pyruvate kinase, 0.1 mM EGTA and 40 mM Tris-HCl, pH 7.4,. Verapamil was added from stock solution in water; cyclosporine A was added from concentrated stock in DMSO such that the final DMSO concentration was 2%; control samples contained 2% DMSO. The decrease in NADH absorbance recorded at 340 nm in a microplate reader (Benchmark Plus, BioRad) was linear between 5 and 20 min. ATPase activity was calculated as described previously [37] and plotted with SigmaPlot 10 (Systat Software, Inc.).

### Circular Dichroism (CD)

CD spectra were recorded at 20°C at a protein concentration of 0.18–0.28 mg/ml in a 0.05 cm cuvette using a thermostated CD spectrophotometer (Olis DSM 1000, USA). Reference and sample buffers contained 5 mM HEPES, pH 7.6, 12 mM NaCl, 2.5% glycerol, 0.05% DDM and 0.25 mM DTT. The α-helical content was determined by the method of Chen et al., (37).

### Scanning Calorimetry (DSC)

Calorimetry was routinely carried out in 20 mM HEPES, pH 7.6, 50 mM NaCl, 10% glycerol, 0.1% DDM and 5.5 mM DTT in 0.13 mL cells at a heating rate of 2 K/min with the VP-Capillary DSC System (MicroCal Inc., GE Healthcare). An external pressure of 2.0 atm was maintained to prevent possible degassing of the solutions on heating. Thermal unfolding was irreversible, as determined by sample cooling and reheating. Heat capacity curves were corrected for instrumental baseline obtained by buffer scans. Separated DSC scans were conducted for buffer containing 1% lipids and no transition was detected in the temperature range of thermal unfolding for the proteins in presence of lipids. DSC data were analyzed with the MicroCal Origin software to obtain the unfolding temperature (*T*_m_) and the total unfolding enthalpy (Δ*H*cal).

### Trypsin digestion and SDS-PAGE

Pgp (5 µg), activated with 1% *E. coli* lipids, was mixed with 2 µl of trypsin (serially diluted in 1 mM HCl from 1.6 to 0.0001 mg/ml). After 15-minute incubation at room temperature, digestion was stopped with 2 µl (5 ug) of trypsin inhibitor (Type I-P from bovine pancreas, Sigma-Aldrich). Samples were mixed with ≥0.3 volumes of sample buffer (125 mM Tris-Cl, pH 6.8, 5% (w/v) SDS, 25% (v/v) glycerol, 0.01% pyronin Y, and 160 mM DTT), incubated for 10 min at RT, then resolved on 10.5-14% polyacrylamide gradient Criterion precast gels (BioRad), and stained with Coomassie Blue.

## Results

### Codon usage bias in *P. pastoris*

We compiled a codon usage table for 30 native genes known to be expressed at high levels in *P. pastoris* [29], [30], [38], [39] (Fig. 1). Although the table was based on a modest number of genes, the resulting codon usage frequencies were quite comparable to those of 263 highly expressed genes in the related yeast *S. cerevisiae* [15]. For example, the most abandoned codon for each amino acid (highlighted in blue, Fig. 1) as well as the codons used at low frequency (<10%, highlighted in orange) were very similar in both species of yeasts (compare columns 3 and 4, Fig. 1). However, codon frequencies were distinctly different from those in the Kazusa or the *Pichia* genome databases, which do not discriminate between poorly and highly expressed genes. Besides five low frequency (<10%) codons seen in the Kazusa database, an additional 18 codons occur only at low frequency among highly expressed genes (compare columns 1 and 2 versus 4, Fig. 1). Thus, codon usage was considerably more stringent for high level compared to low or medium level expression. Also, among highly expressed genes certain high frequency codon preferences were inverted: CAC over CAU (73∶27%) for His, UUC over UUU (67∶33%) for Phe, GAC over GAU (59∶41%) for Asp and GAG over GAA (58∶42%) for Glu (for more details see Fig. 1 legend). Consequently, adoption of codon frequencies seen in highly expressed genes may represent a better choice for optimization of genes for high level expression.

### Optimization of the Pgp gene

Codon frequencies within the 3828 bp coding sequence of the native mouse *mdr3* gene (also called *mdr1a* or *abcb1a)* differed markedly from those of *P. pastoris* highly expressed genes, with pronounced over-representation of yeast low frequency codons and under-representation of yeast preferred and higher frequency codons (see column 5, Fig. 1). In addition, the native gene sequence showed 38 tandem codon repeats, 99 regions of extended secondary mRNA structure (hairpin loops) that can hinder translation, 86 AT-rich or GC-rich regions (up to 10 bases in length), 9 cryptic splice sites, and a GC content of 48% which is somewhat higher than that found in highly expressed *Pichia* genes (45%). These structural elements, along with the codon bias, appeared unfavorable for high-level expression in *P. pastoris*, and our strategy to optimize the *mdr3* sequence was as follows: We omitted all occurrences of the 19 low frequency codons (<8%) and we set the relative frequencies among the remaining codons similar to those of highly expressed genes. We also avoided codon repeats and AT-rich regions, and adjusted the GC content to 45% (balanced to ±10% within a 40 bp window throughout the gene) (Fig. S2B). The resulting gene sequence (“*opti-mdr3*”) is given in Fig. S3 (GenBank JF834158) and the final codon usage is shown in Fig. 1, column 6.

### Functional analysis of Opti-Pgp in *S. cerevisiae*

**Figure 2:**
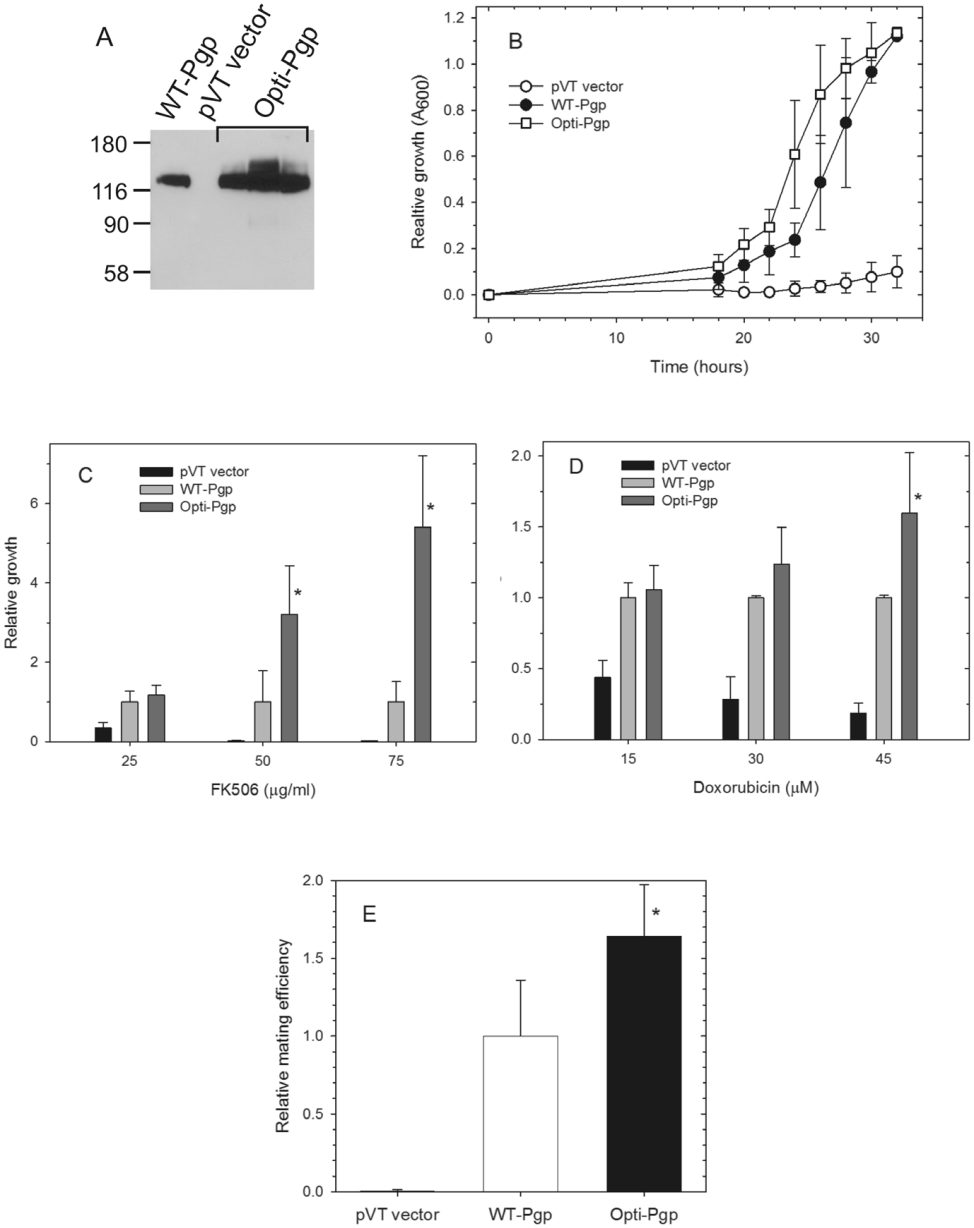
Protein expression levels and *in vivo* biological activity of WT- and Opti-Pgp in *S. cerevisiae*. A) Three independent pVT-*opti-mdr3* clones were transformed into *S. cerevisiae*, microsomal membrane proteins (15 µg) of mass populations resolved on a 10% SDS-gel and the Western blot probed with the Pgp-specific monoclonal C219 antibody (Covance SIG-38710). Mass populations transformed with pVT vector alone or the WT gene served as controls. The positions of the MW protein markers are indicated in kDa. B) Growth resistance to the fungicide FK506 (50 µg/ml) was monitored at A_600_ for wild-type Pgp (WT-Pgp), gene-optimized Pgp (Opti-Pgp) and control pVT vector transformants. Data points represent the mean ± standard deviations of three independent transformants assayed in triplicate in four independent experiments; where not visible, error bars are smaller than the plot symbol. C) Growth of individual mass populations in the absence or presence of increasing concentrations of FK506 (25, 50 and 75 µg/ml) was measured at A_600_ after 25-26 hours and is expressed as growth relative to WT-Pgp. D) Growth resistance in the absence or presence of doxorubicin (15, 30 and 45 µM) was measured relative to WT-Pgp. E) Mating frequency represents the proportion of transformed **a**-type JPY201 cells that formed diploids upon mating with R-type tester cells DC17, followed by plating on minimal medium [34]. Values are expressed as a percentage of the WT frequency ± the standard deviation of four experiments using three independent transformants. Asterisks indicate significant differences between WT- and Opti-Pgp (p<0.05).

Because codon usage of highly expressed genes is so similar in *S. cerevisiae* and *P. pastoris*, we expected our optimization approach to improve expression in both yeasts. For three mass populations of independent *S. cerevisiae* transformations, Pgp-specific signal intensities in Western blots of microsomal membranes indicated that Opti-Pgp transformants expressed the protein at two- to three-fold higher levels than did WT-Pgp transformants (Fig. 2A). This indicated that gene optimization indeed enhanced expression levels in yeast.

Although the optimized gene encodes identical primary amino acid sequence to the WT protein, co-translational effects might cause changes in protein folding [40]. Therefore, it was important to demonstrate that Opti-Pgp retained full biological activity. Procedures to test *in vivo* Pgp function in *P. pastoris* have not been developed, so to take advantage of established biological assays [12], [33], [34] and to examine substrate specificity, we first tested Opti-Pgp function in the yeast *S. cerevisiae*. We previously showed that expression of native Pgp in *S. cerevisiae* confers drug resistance against fungicides [12], [33], [41], so we first measured growth-resistance of mass populations to the macrolide immunosuppressant FK506. In four independent experiments Opti-Pgp transformants grew faster than WT-Pgp in the presence of FK506, i.e. they entered log-phase growth approximately 22 h after inoculation and reached stationary phase at approximately 28 hours, two hours sooner than WT-Pgp (Fig. 2B). Similarly, growth of Opti-Pgp transformants in the presence of the cyclic peptide ionophore valinomycin (80 µg/ml) appeared to be as good as or better than WT-Pgp transformants (data not shown). To better assess potential differences in growth resistance between WT- and Opti-Pgp transformants we grew the cultures in the presence of increasing concentrations of FK506 (Fig. 2C). At concentrations of 25 µg/ml FK506 no difference was evident (pairwise Tukey test comparison p = 0.577) but at the higher concentrations of 50 or 75 µg/ml FK506 Opti-Pgp cultures grew significantly faster than Wt-Pgp (p = 0.025 and 0.003, respectively). Pgp is known to convey multidrug resistance by transporting a wide variety of structurally unrelated compounds. To demonstrate that polyspecificity was maintained in the Opti-Pgp we also measured its ability to confer *S. cerevisiae* with resistance to the anticancer drug doxorubicin. At concentrations of 15 and 30 µM doxorubicin, a pairwise comparison (Tukey test) between WT- and Opti-Pgp revealed no significant difference (p = 0.809 and 0.197) but at the higher concentrations of 45 µM doxorubicin Opti-Pgp cultures grew significantly faster than WT-Pgp (p = 0.034, Fig. 2D). The data demonstrate that Opti-Pgp, like WT-Pgp, transported a range of fungicidal and anticancer drugs. Higher protein expression levels in the Opti-Pgp strains (Fig. 2A) likely accounted for their enhanced drug resistance compared to the WT-Pgp strains.

Pgp also imparts *S. cerevisiae* with the capacity to export **a**-factor mating peptide, permitting diploid formation that can be efficiently measured in a mating assay [12], [33]. Thus we also compared the capacity of Opti-Pgp to restore mating in the sterile *ste6*Δ yeast strain JPY201. Mating frequencies of Opti-Pgp transformants were about 1.5-fold higher than WT-Pgp controls (p = 0.021, Fig. 2E) indicating that Opti-Pgp can export this pheromone more efficiently than WT-Pgp. Together, the results of functionality studies were consistent with higher protein expression, more effective folding and/or more complete trafficking of Opti-Pgp to the cell surface where it executes its biological activity.

### Purification of Opti-Pgp from *P. pastoris*

**Figure 3:**
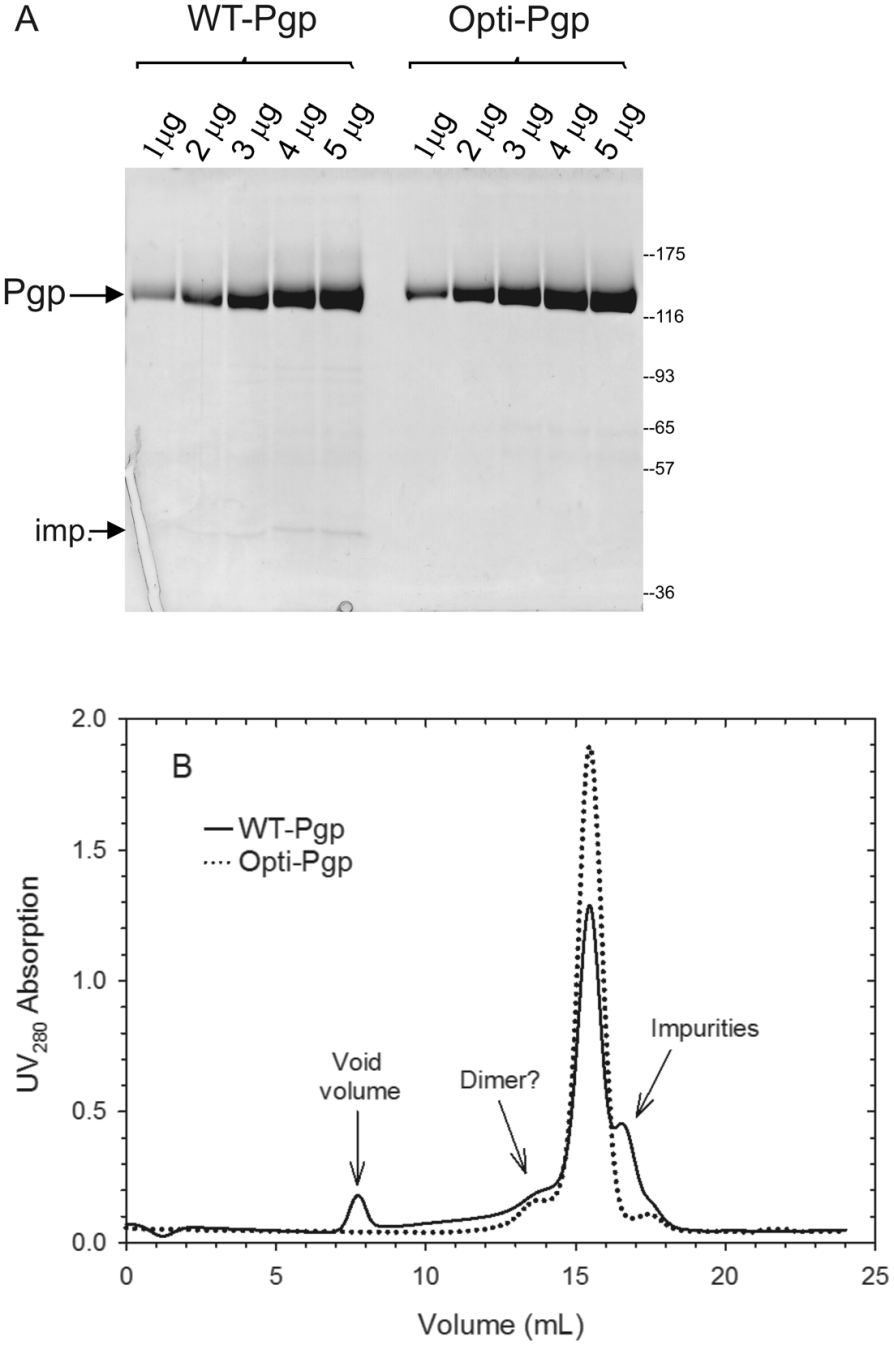
Purification and size exclusion chromatography of WT- and Opti-Pgp from *P. pastoris*. A) Proteins were purified from *P. pastoris* fermentor cultures by chromatography on Ni-NTA and DE52 resin. Increasing amounts of proteins (1 to 5 µg) were resolved on a 10% SDS-gel and stained with Coomassie Blue. The positions of the MW protein markers are indicated in kDa; the protein band labeled “Imp.” (impurities) did not cross-react with the Pgp-specific antibody C219. B) Two milligrams (500 µl) of purified, detergent soluble proteins were loaded on a Superose 6B column and resolved in buffers containing small amounts of detergent (see Materials and Methods). A representative of four independent runs is shown for WT-Pgp (solid line) and Opti-Pgp (dotted line). Molecular mass markers were resolved under identical buffer conditions, the elution volumes were as follows: Blue-dextran (void volume) 6.7 ml, thyroglobulin (669 kDa) 12.4 ml, ferritin (440 kDa) 14.2 ml. aldolase (158 kDa) 15.8 ml, conalbumin (75 kDa) 16.8 ml and ovalbumin (43 kDa) 17.1 ml. The calculated molecular mass of monomeric Pgp (including the His_6_-tag) is 142 kDa, the predicted detergent micelle size for DDM is about 70 kDa.

For large-scale protein production, fermentor cultures of WT- and Opti-Pgp expressing strains of *P. pastoris* were grown and the proteins purified as described in Materials and Methods [13]. Consistently higher yields of purified proteins were obtained from the Opti-Pgp strain (13±3.2 mg per 100 g cells, n = 6) than WT-Pgp (4.3±1.6 mg per 100 g cells, n = 3) (Table 1). Perhaps as a result of yield, purified Opti-Pgp preparations also exhibited lower residual contaminant levels than the 5–10% seen in WT-Pgp preparations on Coomassie-stained gels (labeled “imp.” in Figs. 3A and 8) and on size exclusion chromatography (SEC) (Fig. 3B). WT-Pgp preparations showed a peak at the void volume of the column (Fig. 3B, solid line) that was not seen with Opti-Pgp (dotted line) suggesting that the latter protein is less prone to aggregation. In both cases the major protein peak appeared monomeric with an elution volume (15.3 mL) indicating an apparent size of approximately 200 kDa, and a minor peak at 13.5 mL consistent with Pgp oligomer [42]. Thus, gene-optimization improved the quality of the purified protein, as collectively evidenced by the higher yield and purity of Opti-Pgp preparations, its monodispersity, and its resistance to aggregation.

**Table 1:** Comparison of WT- and Opti-Pgp.

	WT-Pgp	Opti-Pgp
**Yield per** **100 g cells**	4.3±1.6 mg	13.0±3.2 mg
**Maximal** **ATPase activity** **(µmol min^−1^ mg^−1^)** 1)	1.8±0.24	2.1±0.28
**Half-maximal stimulation by Verapamil** **(µM)** 2)	9.1±4.6	4.2±2.2
**Half-maximal inhibition by cyclosporine A** **(µM)** 2)	0.98±0.24	1.1±0.26

1) Average and standard deviations (n>30) from at least three independently purified preparations.

2) Concentrations required for half-maximal stimulation or half-maximal inhibition of ATPase activity were calculated from the fits shown in figures 5 and 6, respectively. Standard deviations are given for individual fits from three independent experiments.

### ATPase activity of purified Opti-Pgp

**Figure 4:**
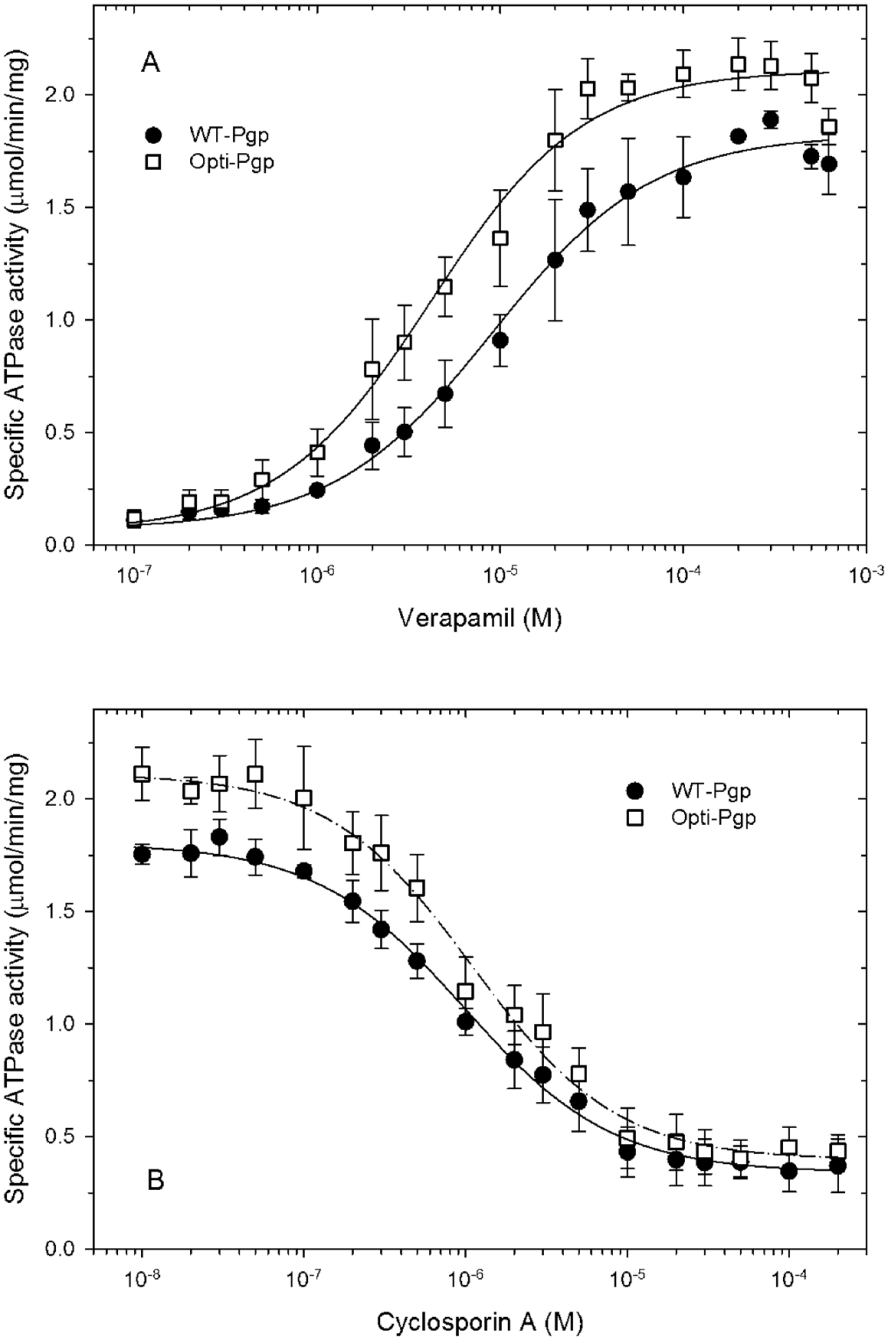
Stimulation and inhibition of ATPase activity. A) The ATPase activity of purified WT- and Opti-Pgp was assayed in the presence of increasing concentrations of verapamil. The solid lines are non-linear regression fits to the equation f = d+(a*x^b^/(c^b^+x^b^)), where d is the activity in the absence of verapamil (basal activity), a is the maximum verapamil-stimulated activity, b is the Hill coefficient, c is the concentration for half-maximal stimulation, and × is the concentration of verapamil. No cooperativity was observed with Hill coefficients close to 1.0 (0.998 and 1.05, respectively). Each data point represents the mean from at least 3 independent experiments (from three different protein purifications) ± standard deviation. B) Purified proteins were assayed in the presence of 150 µM verapamil to maximally stimulate ATPase activity but with increasing concentrations of the inhibitor cyclosporine A. The solid lines are non-linear regression fits to the equation f = a-(e*y^b^)/(c^b^+y^b^)), where e is the maximum inhibition, and y is the concentration of cyclosporine A. No cooperativity was observed with Hill coefficients close to 1.0 (0.95 and 0.98, respectively).

ATPase activity of Opti-Pgp in the presence of 150 µM verapamil was 2.1±0.28 µmol/min/mg (n>30) and was somewhat higher than WT-Pgp (1.8±0.24 µmol/min/mg, n>30), consistent with the low-level impurities and aggregation products present in WT-Pgp preparations (Fig. 3A and B). The half-maximal stimulatory concentrations for verapamil were 4.2 and 9.1 µM for Opti- and WT-Pgp, respectively (Fig. 4A), not significantly different in the two tail test (p = 0.24). Inhibition of the verapamil-stimulated ATPase activity by the immunosuppressant cyclosporine A was also comparable for the two proteins, with half-maximal inhibition seen at 0.98 µM and 1.1 µM for Opti- and WT-Pgp, respectively (p = 0.588, Fig. 4B). The enzymatic data indicate unaltered affinities for substrates and inhibitors in the purified proteins.

### CD spectroscopy

**Figure 5:**
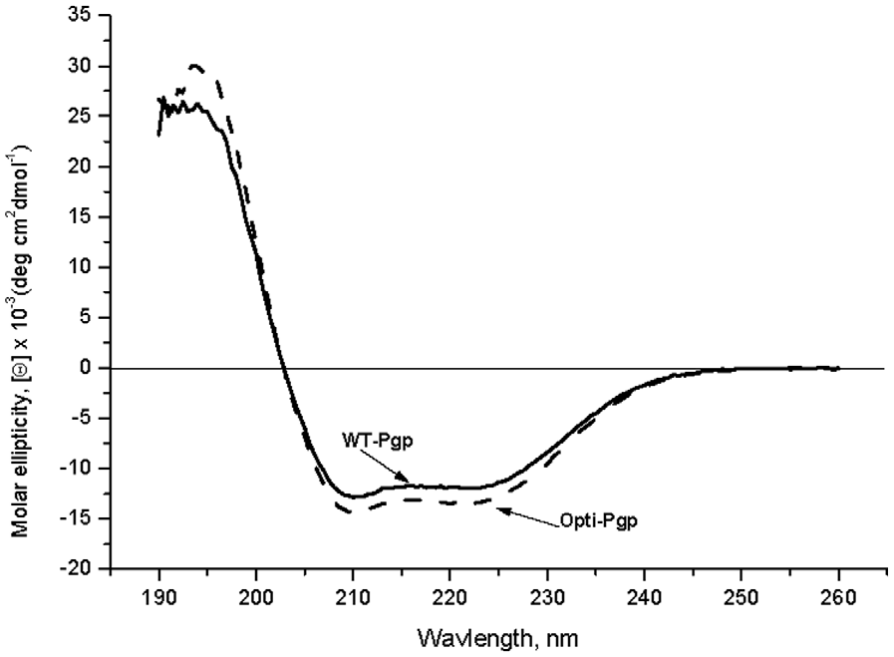
CD spectra of WT- and Opti-Pgp. CD spectra of the purified proteins were recorded after buffer exchange by size-exclusion chromatography (peak fractions from Fig. 3B). Protein concentrations were determined by UV spectroscopy, as well as the colorimetric BCA protein assay using BSA as a standard; the two assays gave essentially the same results. Each spectrum represents an average of 10 scan from three different protein preparations. Molar ellipticity values were calculated according to [Θ]  =  Θ (100× MRW/*lc*), where Θ is the measured ellipticity in degrees, MRW is the molecular weight of Pgp (141,000 g/mol), *l* is the path length in centimeters, and *c* is the concentration of the protein in grams per liter [43].

To monitor potential differences in secondary structure, WT- and Opti-Pgp were investigated by far-UV CD (Fig. 5). The shape of the curves was essentially identical, as was the size of the peak near 220 nm, suggesting the presence of a significant amount of α-helicity. In fact, the α-helical content was estimated to be approximately 41% for WT- and 46% for Opti-Pgp using the method of Chen *et al.* [43]. These values are very close considering that accurate protein concentration determination is critical for these estimates.

### Thermal Unfolding of WT- and Opti-Pgp

**Figure 6:**
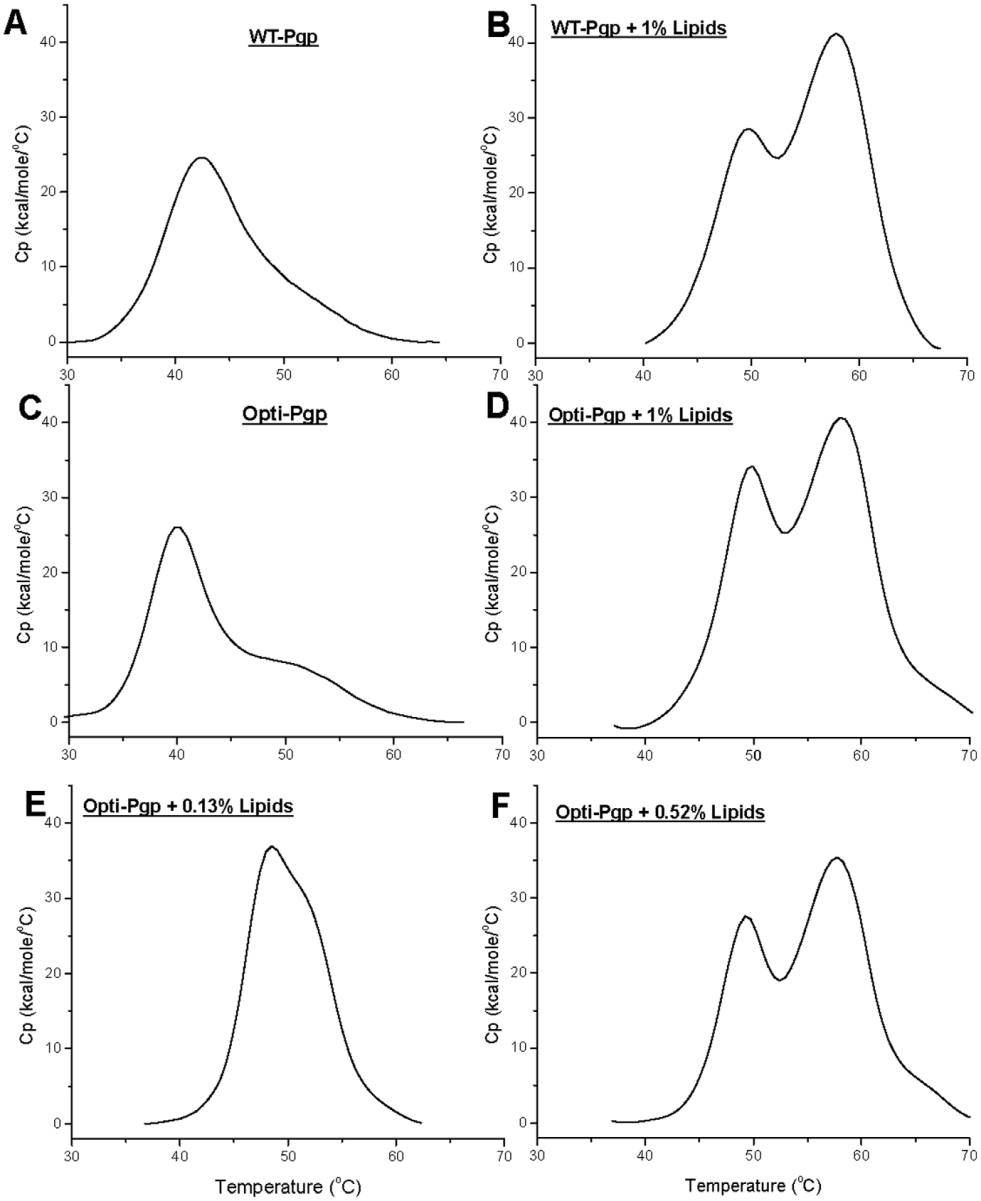
Differential Scanning Calorimetry of WT- and Opti-Pgp. Purified proteins were exchanged into buffer containing a defined DDM concentration (as in Fig. 3B), and the temperature dependence of the molar heat capacity recorded; protein concentrations ranged between 0.45–0.78 mg/ml for WT-Pgp and 0.58–0.78 mg/ml for Opti-Pgp, respectively. Panels A and C: no lipid added. Panels B and D: Proteins were preincubated with 1% (w/w) *E. coli* lipid (lipid to protein ratio of 16:1, w/w) for 15 min at RT followed by 30 s bath sonication as described [13]. Panels E and F: Opti-Pgp was preincubated with 0.13% or 0.52% (w/w) *E. coli* lipid (lipid to protein ratios of 2.2:1 and 8.4:1, w/w)). Control samples containing the same amount of lipid had no detectable transition in the temperature range of protein unfolding.

**Figure 7:**
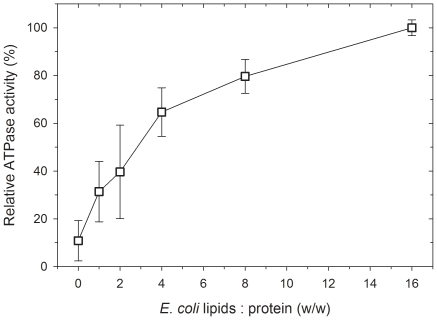
Lipid dependence of ATPase activity. ATP hydrolysis of Opti-Pgp was assayed after activation with increasing concentrations of *E. coli* lipids as described in Materials and Methods. Averages ± range of two independent experiments are given. 1% lipids added correspond to a lipid: protein ratio of 16:1.

Thermal unfolding was monitored by DSC to directly probe protein stability and cooperativity of unfolding. At the least, a detectable DSC transition supports the presence of a folded, cooperative tertiary structure. Comparison of the upper and middle panels of Fig. 6 shows that the unfolding T_m_ and the shape of the unfolding transitions are essentially the same for WT- and Opti-Pgp, whether in detergent solution (panels A and C) or after addition of 1% lipids (panels B and D), i.e. under conditions giving maximum ATP hydrolysis rates [13]. The presence of lipid shifted the T_m_ from ∼40°C (with a minor transition apparent at ∼50°C) to higher temperatures, with the concurrent appearance of two clear transition maxima near 50°C and 58°C (Table 1). The significant increase in the total unfolding enthalpy ΔH_cal_ for both proteins upon lipid addition indicated improved stability and suggested an increase in stable tertiary structure of Pgp when surrounded by lipids. Further measurements of the thermal unfolding of Opti-Pgp at limiting lipid concentrations (panels E and F, Fig. 6) demonstrated that the T_m_ and ΔH_cal_ increased gradually, with a single but asymmetric peak seen at 0.13% lipid while the second transition appeared at lipid concentrations of ≥0.52%. Similarly, verapamil-stimulated ATPase activity of Opti-Pgp showed an increase from 11% in the absence of lipids to 40% and 80% in the presence of 0.13% and 0.52% lipid (Fig. 7). The observation of two defined transitions in the presence of lipid is consistent with the presence of at least two structural domains of different stabilities which, in the absence of lipid, may be energetically equivalent or may not manifest as distinct domains. These are only two possible explanations; others may be equally feasible. Taken together, the thermal unfolding profiles are consistent with a folded protein that gains stability and, most likely, structure as a function of lipid concentration.

### Tryptic digestion profiles of purified WT- and Opti-Pgp

**Figure 8:**
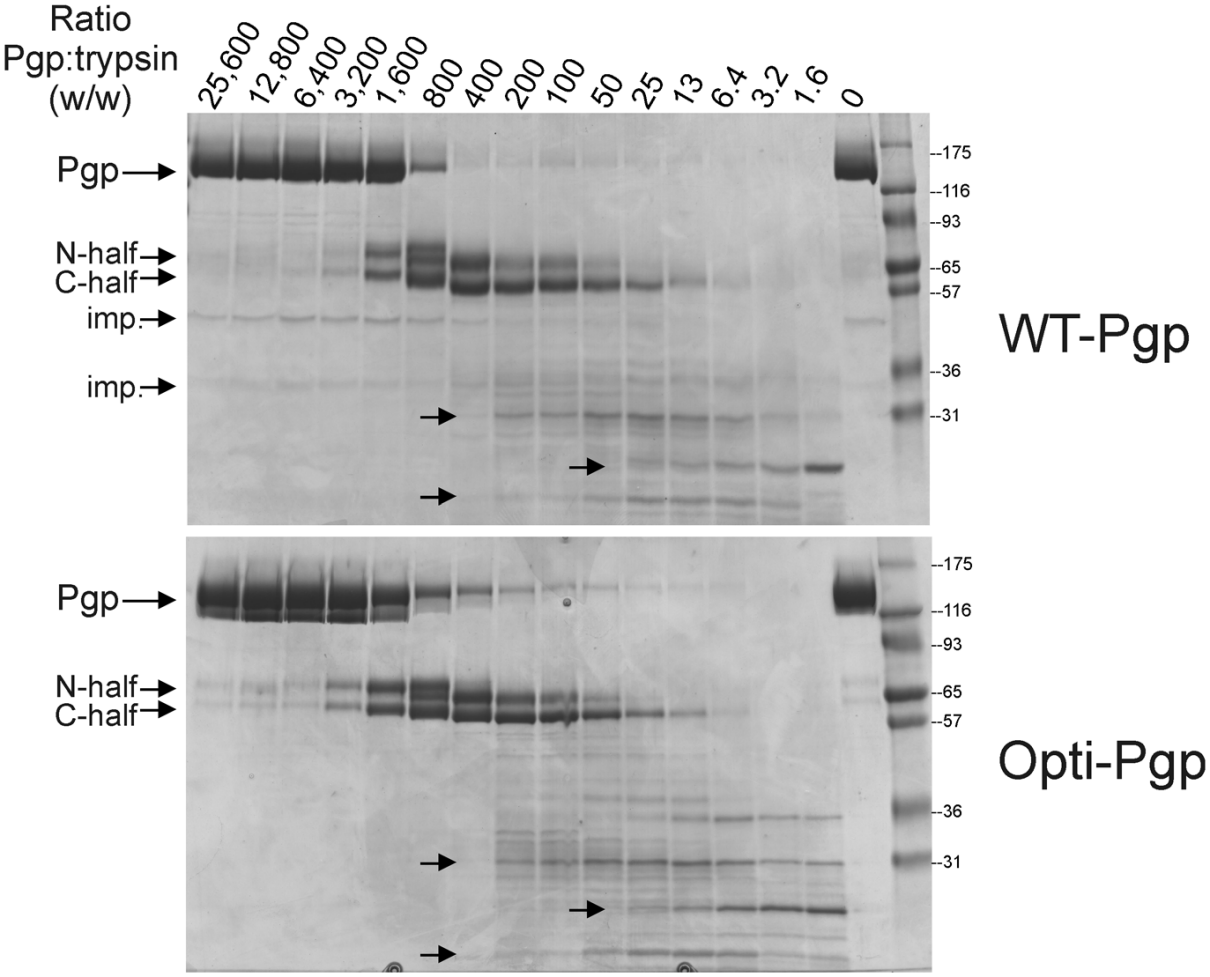
Determining the sensitivity of WT- and Opti-Pgp to trypsin. Five µg of purified lipid-activated proteins were incubated with increasing concentrations of trypsin. Samples were resolved on 10.5–14% gradient gels and stained with Coomassie-Blue. The positions of the MW protein markers are indicated in kDa. Arrows indicate the position of the full-length proteins (Pgp), the N-terminal or C-terminal half size proteins, and the position of major tryptic fragments; Imp., impurities.

To disclose subtle differences in folding between WT- and Opti-Pgp, we compared their relative susceptibilities to limited proteolysis by trypsin. Figure 8 shows the disappearance of the Pgp band as a function of trypsin; the concentration required for 50% degradation (expressed here as the ratio of Pgp:trypsin) was the same for WT- and Opti-Pgp. Coincident appearance of the N- and C-terminal half fragments produced by the action of trypsin at the first cleavage sites in the linker region [44] as well as of smaller fragments (36 kDa, 31 kDa and smaller, arrows) at a given concentration of trypsin argues that the principle cleavage sites were equally accessible in the two proteins. This result implied that the two had similar tertiary structures, which was completely consistent with the CD and DSC results.

## Discussion

As a eukaryotic expression system, *P. pastoris* has many advantages, such as efficient protein folding, membrane targeting, proteolytic processing, disulfide formation and glycosylation [45]. It is a cost-effective system that provides high biomass in fermentor cultures and thus greater amounts of protein per culture volume than any other system, and therefore proved an ideal choice for Pgp production for X-ray crystallography and functional studies [11], [12], [37], [46], [47], [48], [49], [50]. Still, as for any membrane protein, production of pure protein for biophysical and enzymological study is a relentless challenge and any improvements in yield, quality and stability of the protein will greatly facilitate downstream analysis.

To maximize protein expression at the translational level we optimized codon usage in the Pgp gene (mouse *mdr3*) according to codon frequency found among highly expressed *P. pastoris* genes, and we also removed mRNA instability motifs and secondary structure that may impair translation [51]. The main purpose of this study was to rigorously analyze the function of gene-optimized “Opti-Pgp” *in vivo* and at the purified protein level to detect any potential differences in function or solution structure, if any, compared to WT-Pgp. Opti-Pgp was expressed at two- to three-fold higher levels and was fully able to convey *in vivo* drug resistance against a broad range of anticancer drugs and fungicides in the related *S. cerevisiae* yeast (Fig. 2). Indeed the growth resistance profiles together with the enhanced capacity of Opti-Pgp to export **a**-factor mating peptide suggested that cotranslational folding and/or trafficking to the cell surface was improved compared to WT-Pgp. Gene-optimization increased Pgp protein production from *P. pastoris* by about three-fold. ATP hydrolysis by the purified protein was strongly stimulated by verapamil (∼15-fold) and inhibited by cyclosporine A with binding affinities indistinguishable from WT-Pgp (Fig. 4, Table 1). Moreover, ATP hydrolysis rates were enhanced (∼1.2-fold) likely due to the higher purity and/or stability of Opti-Pgp preparations. SEC of Opti-Pgp samples that were frozen and thawed once showed a symmetrical peak with a retention volume corresponding to monomeric protein, and no aggregated protein was detected at the void volume of the column in contrast to WT-Pgp samples (Fig. 3). The functionality data, together with the higher yield and purity, as well as its monodispersity in SEC and lower background protein aggregates in crystallization trays (not shown) suggest that Opti-Pgp will be a most valuable tool for future biophysical studies requiring large amounts of high quality protein.

These important findings were extended further by analyzing purified Pgp conformation by CD, DSC and limited proteolysis. WT- and Opti-Pgp showed very similar CD profiles suggesting an α-helical content of about 41–46% in DDM solution [43], a value somewhat lower than the ∼60% α-helical content calculated from X-ray structures solved in the same detergent [11]. Higher flexibility of the protein in solution and/or the absence of cholate, transport substrate, nucleotide, inhibitors or additives necessary for crystallization may account for this lower helicity value [52], [53], [54]. We previously demonstrated a strong dependence of Pgp ATPase activity on the presence of lipid [13], indicating that lipids promote an active conformation of Pgp, possibly through interactions with the hydrophobic TMDs. Here we show for the first time that the presence of 1% *E. coli* lipid increased the thermal stability of the protein as indicated by a shift in T_m_ from ∼40°C to 49°C, as well as a significant increase in the total unfolding enthalpy ΔH_cal_ of both WT- and Opti-Pgp (Fig. 6, Table 2). Strikingly, a distinct second unfolding transition appeared at ∼58°C suggesting sequential unfolding of at least two domains in the protein [55], [56]. It is tempting to assign the higher transition to unfolding of the TMDs which, under these conditions, are expected to reside within the hydrophobic core of the lipid bilayer. This environment may promote the acquisition of a more cooperative and/or more folded structure by providing better aqueous solvent exclusion for the TMDs than detergent, and/or there may be specific lipid-protein interactions which would thermodynamically favor a more folded structure. Other explanations for TMD stabilization are also possible [57], [58]. Titration of Opti-Pgp with lipid showed that the lipid-dependent changes in T_m_ occurred progressively, with an intermediate T_m_ seen at 0.13% lipid (48°C) and two distinct T_m_ maxima resolving at lipid concentrations ≥0.52% (Fig. 6C–F). The increase in thermal stability was paralleled by an increase in ATPase activity with increasing lipid concentrations (Fig. 7). Together, the data suggest that an increase in stable ternary structure over the entire Pgp molecule may be responsible for the robust ATPase activity seen when the protein is surrounded by saturating lipid molecules. However, phospholipids also serve as transport substrates of Pgp [59] and we cannot exclude the possibility that some lipid-substrate molecules bound to the drug binding site may promote folding in the manner of chemical chaperones, in addition to hydrophobic interactions at the protein-lipid interface [60].

**Table 2:** Thermal unfolding parameters of WT- and Opti-Pgp.

Sample	Addedlipids	Unfolding temperature(°C)		ΔH_cal_(kcal/mol)	n b
		T_1_ a	T_2_ a		
WT-Pgp	None	43.0±1.6	ND	264±87	5
	1% lipid	50.4±0.9	57.8±0.1	518±4.2	2 c
Opti-Pgp	None	42.7±1.7	ND	264±67	11 d
	1% lipid	49.3±1.0	58.7±0.5	567±33	5

a Temperatures corresponding to the two maxima of the unfolding profiles seen in Fig. 6.

b Number of independent experiments.

c Averages ± range are given.

d Experiments were routinely conducted in 20 mM HEPES, pH 7.6, 50 mM NaCl, 10% glycerol, 0.1% DDM and 5.5 mM DTT. Four experiments were conducted in buffers containing 40 mM imidazole, and three experiments were conducted with reduced glycerol (5% instead of 10% glycerol); no significant differences in the T_m_ or ΔH_cal_ were observed under those conditions.

Previously, human Pgp single-nucleotide polymorphisms (SNPs) that introduce rare codons were suggested to alter the structure of substrate and inhibitor interaction sites by affecting the timing of cotranslational folding and membrane insertion [40], [61], [62], [63]. In these studies, the human *MDR1* haplotype consisting of the synonymous polymorphisms C3435T (Ile1145) and C1236T (Gly412) in combination with G2677T, which changes Ala893 to Ser led to reduced Pgp affinity for verapamil and the inhibitor cyclosporine A. Additionally, this haplotype altered susceptibility of the protein to trypsin cleavage [40]. These studies suggested that the tertiary structures of wild-type and the haplotype Pgp differed, which may affect the pharmacokinetics and efficacy of cancer drug treatment [61]. Because of the potential impact of even subtle conformational changes, it was important to confirm that Opti-Pgp retained both substrate specificity (see Figs. 1 and 3) and tertiary structure. Trypsin cleavage sites appeared equally accessible in WT- and Opti-Pgp (Fig. 8), suggesting that the two proteins indeed have a similar folded state. This was also corroborated in our DSC study by their similar unfolding temperatures and enthalphies in the absence or presence of lipids (Fig. 6A–D, Table 2). Interestingly, two of these haplotype codons occur in the homologous positions of the native mouse gene: Ile1141 (ATT) and Ser889 (TCT). It may be noted that ATT and TCT actually represent preferred codons in *Pichia* yeast (Fig. 1), in contrast to codons found in human genes. Thus, introduction of these SNPs during codon-optimization of the mouse (or human) gene for *Pichia* would not be expected to affect cotranslational folding and membrane insertion of Pgp in yeast expression systems.

Finally it is appropriate to comment on the superior optimization procedure proposed in this study. Previous gene optimization procedures aimed to adjust codon usage of the heterologous gene sequence to that of the *P. pastoris* host either by replacing codons with low usage percentage (<15%) by those with higher usage frequency [21], [64], [65], or, more recently, by simply changing all codons to the most frequently used synonymous codon [66], [67]. Codon analyses, including those offered by commercial sources (e.g. GeneArt, GenScript) were commonly based on the Kazusa codon usage database (http://www.kazusa.or.jp/codon/). Neither the Kazusa database, currently containing 137 coding sequences (CDS's), nor the more complete codon usage table of the *P. pastoris* ORFeome with 5,313 CDS's that was recently obtained by genome sequencing [23], [29], discriminates between poorly and highly expressed genes. But codon usage in *P. pastoris* (and in *S. cerevisiae*) appears significantly more stringent in highly expressed genes, as evident from the larger number of low-frequency codons (Fig. 1). Furthermore, there are inverted preferences for certain yeast preferred and higher frequency codons (see Fig. 1 legend), suggesting that preferred codons assigned in the Kazusa database may not always represent the best codon choice for high level expression [19], [21], [68]. The new approach in this study was not only to omit 19 rare codons (<8% frequency) but to completely harmonize the frequency of codons to those of highly expressed *P. pastoris* genes, and so to maximize translational efficiency by emulating the host's evolutionarily determined codon usage strategy [51], [69].

In conclusion, these studies provide evidence that substrate specificity and folding were preserved in the gene-optimized Pgp expressed in *P. pastoris*. Together with transport function, higher protein yield and purity warrant the use of this protein for biophysical studies. Furthermore, the successful gene optimization approach described here may provide a basis for yeast expression of other ABC transporters and membrane proteins, especially in those cases in which poor expression of the native gene have precluded purification efforts [35]. Indeed, preliminary expression analyses of poorer expressers than the mouse Pgp, e.g. the human Pgp (MDR1) or the Cystic Fibrosis Conductance Regulator (CFTR), a protein notorious for its low expression and high turnover in cells [70], suggest that expression levels are increased at least 5-fold compared to the respective WT proteins (purification trials and functional analyses are currently in progress). Finally, gene synthesis concurrent with gene optimization may offer a cost effective alternative for expression of proteins identified from genome sequencing projects for which a physical cDNA is not yet available.

## Supporting Information

Table S1 30 native *P. pastoris* genes known to be highly expressed [26], [27], [28].
(DOC)

Figure S1 **Restriction sites and GC content of the** ***Opti-Pgp*** **gene.** A) The 3,828 bp coding sequence (CDS) of mouse *mdr3* is shown with unique restriction enzyme sites; *SacII*, *NruI*, *AvrII*, *SalI* and *SpeI* are not present in the Wt sequence, and the gene is flanked by *BstBI* and *XhoI* sites. B) The plot shows the GC content analyzed with GeneOptimizer (GeneArt, Germany) of the *Opti-Pgp* gene in a 40 bp window centered at the indicated nucleotide position.
(TIF)

Figure S2 **Cloning strategy for pLIC-H_6_ vector and expression in** ***P. pastoris*** **.** Schematic representation of the expression construct for ligation-independent cloning (LIC) using the pLIC-H_6_ vector described in [31]. Single-stranded overhangs, produced by the 3′ to 5′ exonuclease reactivity of T4 DNA polymerase in the presence of dGTP and dCTP, are shown for the PCR-amplified gene (top) and the corresponding counterparts in the vector (bottom), respectively. After cloning, the pLIC-H_6_ plasmid encodes a protein bearing a C-terminal His_6_ tag. In addition, the vector contains Kozak-like bases in the region around the ATG start codon (positions -3 and +1) important for high-level expression in *P. pastoris* [31]. Integrity of the CDS was confirmed by DNA sequencing. The resulting plasmids pLIC-*mdr3*-H_6_ and pLIC-*opti-mdr3*-H_6_ were transformed into *P. pastoris* strain KM71H and selected on 100 μg/ml Zeocin as described [35].
(TIF)

Figure S3 **Amino acid and nucleotide sequence alignment of wild-type** ***mdr3*** **and** ***Opti-mdr3.***
(DOC)
