# Supplementary material for: A Gene Optimization Strategy that Enhances Production of Fully Functional P-Glycoprotein in Pichia pastoris
Source: PLoS One. 2011 Aug 3;6(8):e22577. doi: 10.1371/journal.pone.0022577 (PMC3149604; doi:10.1371/journal.pone.0022577)
Supplement: Table S1 — 30 native P. pastoris genes known to be highly expressed [26], [27], [28]. (DOC) [file pone.0022577.s001.doc]

**Supporting Information**

**Table S1: 30 native *P. pastoris* genes known to be highly expressed** .

| **GeneBank** | **GeneName** | **Description** |
| --- | --- | --- |
| Pipas_chr1-3_0104 | ACO1 | Aconitase, required for the tricarboxylic acid (TCA) cycle |
| Pipas_chr2-1_0767 | ACS1 | Acetyl-coA synthetase isoform |
| Pipas_chr4_0821 | AOX1 | Alcohol oxidase |
| Pipas_chr3_0069 | CAT2 | Carnitine acetyl-CoA transferase present in both mitochondria and peroxisomes |
| Pipas_chr2-2_0127 | CCP1 | Mitochondrial cytochrome-c peroxidase |
| Pipas_chr2-1_0769 | CDC19 | Pyruvate kinase |
| Pipas_chr2-2_0131 | CTA1 | Catalase A, breaks down hydrogen peroxide in the peroxisomal matrix formed by acyl-CoA oxidase (Pox1) |
| Pipas_chr3_0082 | ENO1 | Enolase I, a phosphopyruvate hydratase that catalyzes the conversion of 2-phosphoglycerate |
| Pipas_chr1-1_0072 | FBA1 | Fructose 1,6-bisphosphate aldolase, required for glycolysis and gluconeogenesis |
| Pipas_chr3_0932 | FDH1 | NAD(+)-dependent formate dehydrogenase, may protect cells from exogenous formate |
| AF066054 | FLD1 | Formaldehyde dehydrogenase |
| Pipas_chr1-1_0107 | GDH3 | NADP(+)-dependent glutamate dehydrogenase |
| Pipas_chr3_0826 | GPM1 | Tetrameric phosphoglycerate mutase |
| Pipas_chr3_0579 | GUT2 | Mitochondrial glycerol-3-phosphate dehydrogenase |
| Pipas_chr1-4_0130 | HSP82 | Heat shock protein Hsp90 |
| Pipas_chr1-4_0338 | ICL1 | Isocitrate lyase, catalyzes the formation of succinate and glyoxylate from isocitrate |
| Pipas_chr1-1_0432 | ILV5 | Acetohydroxyacid reductoisomerase |
| Pipas_chr2-1_0140 | Kar2 | ATPase involved in protein import into the ER, also acts as a chaperone to mediate protein folding |
| Pipas_chr2-1_0238 | MDH1 | Mitochondrial malate dehydrogenase, catalyzes interconversion of malate and oxaloacetate |
| Pipas_chr2-1_0160 | MET6 | Cobalamin-independent methionine synthase, involved in amino acid biosynthesis |
| Pipas_chr4_0844 | PDI | Protein disulfide isomerase, multifunctional protein resident in the endoplasmic reticulum lumen |
| Pipas_chr1-4_0292 | PGK1 | 3-phosphoglycerate kinase |
| Pipas_chr1-4_0569 | PIL1 | Primary component of eisosomes |
| Pipas_chr1-3_0068 | RPP0 | Conserved ribosomal protein P0 similar to rat P0, human P0, and E. coli L10e |
| Pipas_chr3_0230 | SSA3 | ATPase involved in protein folding and the response to stress |
| Pipas_chr3_0731 | SSB2 | Cytoplasmic ATPase that is a ribosome-associated molecular chaperone |
| Pipas_chr3_0365 | SSC1 | Mitochondrial matrix ATPase |
| Pipas_chr2-1_0437 | TDH3 | Glyceraldehyde-3-phosphate dehydrogenase (GAP), isozyme 3, involved in glycolysis and gluconeogenesis |
| Pipas_FragB_0052 | TEF2 | Translational elongation factor EF-1 alpha |
| Pipas_chr4_0038 | YEF3 | Translational elongation factor 3 (TEF3), stimulates the binding of aminoacyl-tRNA (AA-tRNA) to ribosomes |
