# Supplementary figures and images for: A Gene Optimization Strategy that Enhances Production of Fully Functional P-Glycoprotein in Pichia pastoris
Source: PLoS One. 2011 Aug 3;6(8):e22577. doi: 10.1371/journal.pone.0022577 (PMC3149604; doi:10.1371/journal.pone.0022577)

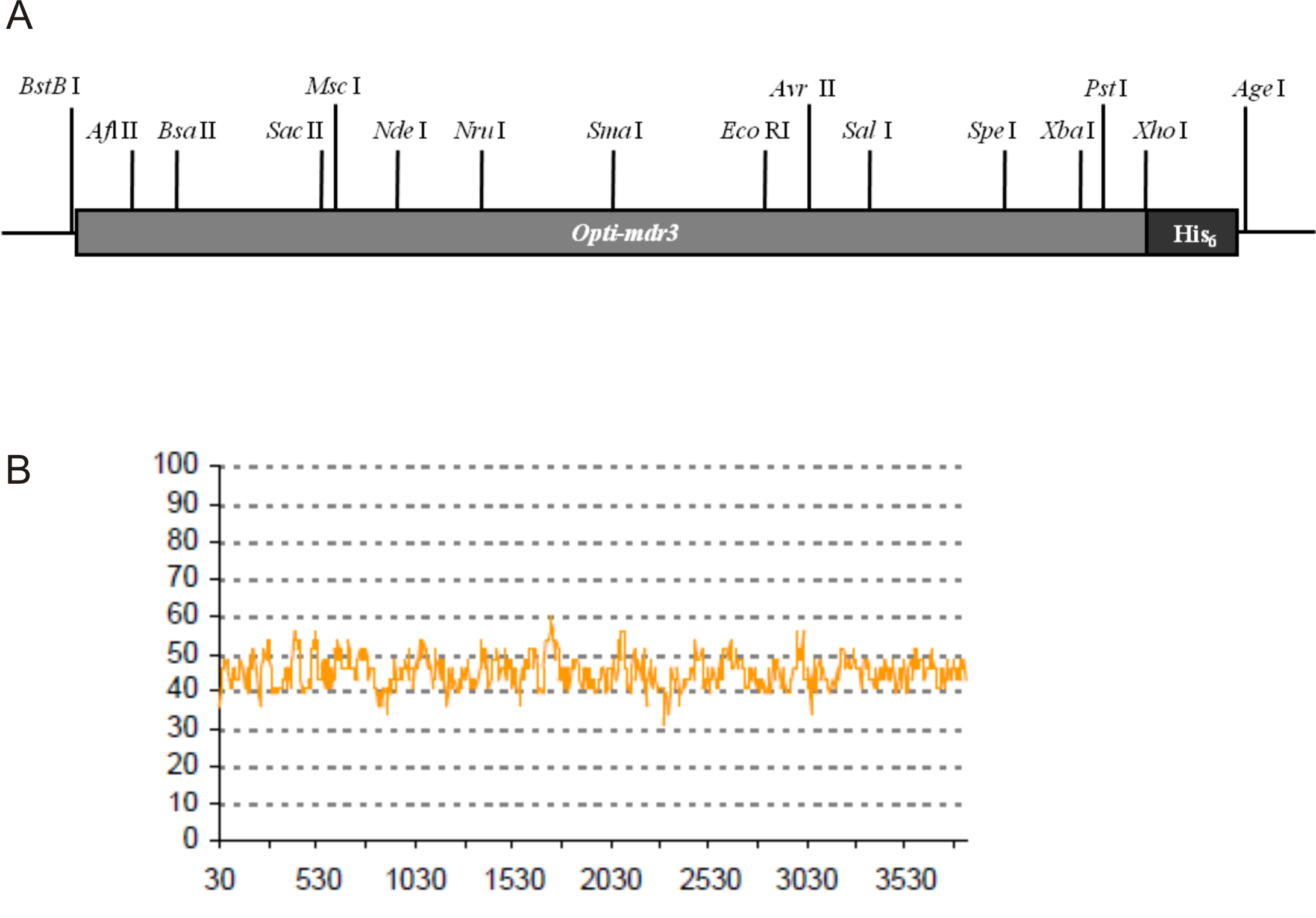

Supplement: Figure S1 — Restriction sites and GC content of the Opti-Pgp gene. A) The 3,828 bp coding sequence (CDS) of mouse mdr3 is shown with unique restriction enzyme sites; SacII, NruI, AvrII, SalI and SpeI are not present in the Wt sequence, and the gene is flanked by BstBI and XhoI sites. B) The plot shows the GC content analyzed with GeneOptimizer (GeneArt, Germany) of the Opti-Pgp gene in a 40 bp window centered at the indicated nucleotide position. (TIF) [file pone.0022577.s002.tif]

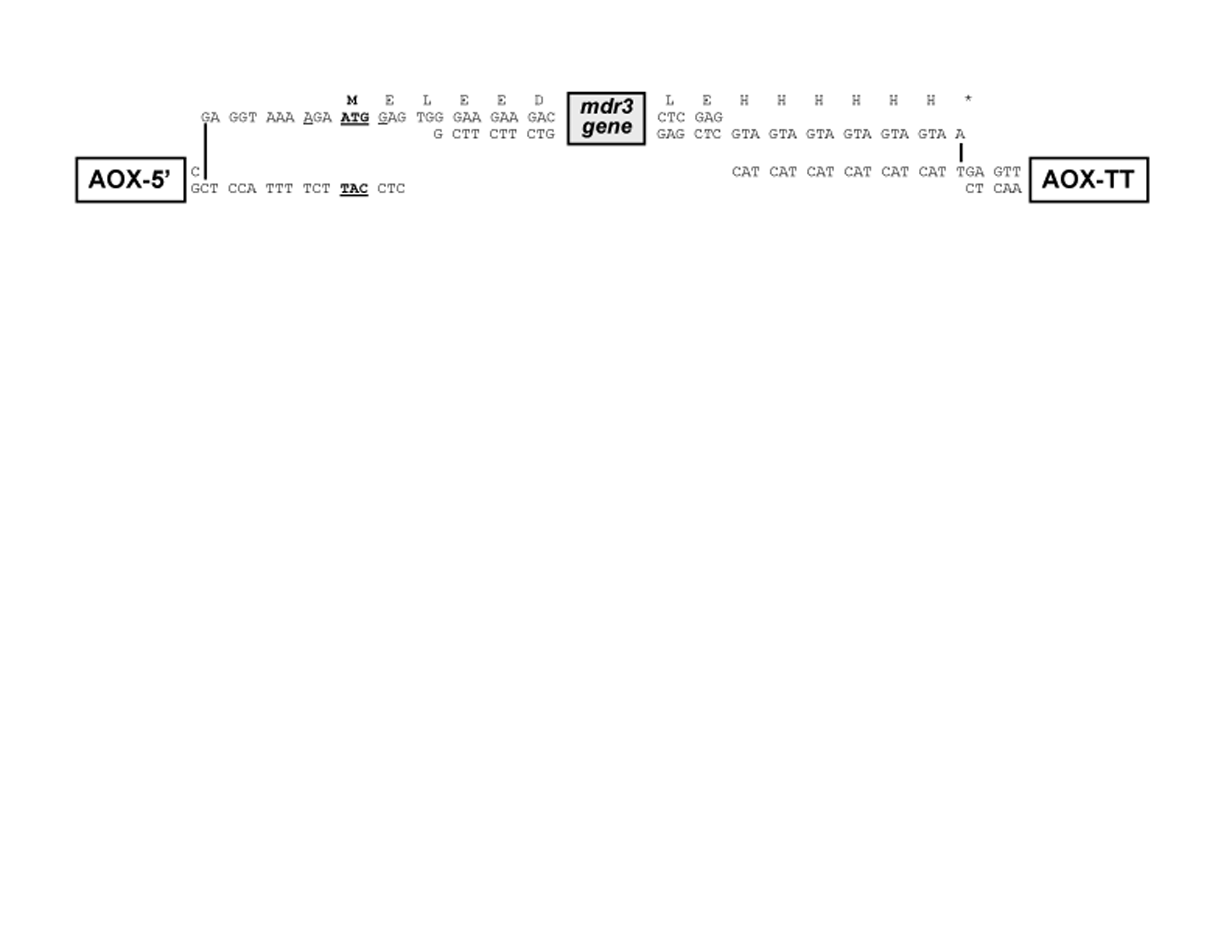

Supplement: Figure S2 — Cloning strategy for pLIC-H6 vector and expression in P. pastoris . Schematic representation of the expression construct for ligation-independent cloning (LIC) using the pLIC-H6 vector described in [31]. Single-stranded overhangs, produced by the 3′ to 5′ exonuclease reactivity of T4 DNA polymerase in the presence of dGTP and dCTP, are shown for the PCR-amplified gene (top) and the corresponding counterparts in the vector (bottom), respectively. After cloning, the pLIC-H6 plasmid encodes a protein bearing a C-terminal His6 tag. In addition, the vector contains Kozak-like bases in the region around the ATG start codon (positions -3 and +1) important for high-level expression in P. pastoris [31]. Integrity of the CDS was confirmed by DNA sequencing. The resulting plasmids pLIC-mdr3-H6 and pLIC-opti-mdr3-H6 were transformed into P. pastoris strain KM71H and selected on 100 μg/ml Zeocin as described [35]. (TIF) [file pone.0022577.s003.tif]
