# Supplementary material for: A Gene Optimization Strategy that Enhances Production of Fully Functional P-Glycoprotein in Pichia pastoris
Source: PLoS One. 2011 Aug 3;6(8):e22577. doi: 10.1371/journal.pone.0022577 (PMC3149604; doi:10.1371/journal.pone.0022577)
Supplement: Figure S3 — Amino acid and nucleotide sequence alignment of wild-type mdr3 and Opti-mdr3. (DOC) [file pone.0022577.s004.doc]

**Figure S3: Amino acid and nucleotide sequence alignment of wild-type *mdr3* and *Opti-mdr3.***

**M E L E E D L K G R A D K N F S K M G K**

**WT 1 ATG GAG TTG GAA GAA GAC CTT AAG GGA AGA GCC GAC AAG AAC TTC TCC AAG ATG GGT AAG**

**Opti 1 ATG GAA CTT GAA GAG GAC CTT AAG GGA AGA GCA GAC AAG AAC TTC TCA AAG ATG GGC AAA**

**K S K K E K K E K K P A V S V L T M F R**

**61 AAG TCC AAG AAG GAG AAG AAG GAA AAG AAG CCA GCA GTT TCT GTG TTG ACC ATG TTC AGA**

**61 AAG AGT AAA AAG GAG AAG AAA GAA AAG AAA CCA GCA GTC AGT GTG CTT ACA ATG TTT CGT**

**Y A G W L D R L Y M L V G T L A A I I H**

**121 TAT GCT GGT TGG TTG GAC CGT TTG TAC ATG TTA GTT GGT ACT TTG GCT GCC ATT ATT CAC**

**121 TAT GCA GGT TGG CTG GAC AGG TTG TAC ATG CTG GTG GGA ACT CTG GCT GCT ATT ATC CAT**

**G V A L P L M M L I F G D M T D S F A S**

**181 GGT GTC GCC TTG CCT TTA ATG ATG CTG ATC TTT GGT GAT ATG ACT GAC TCC TTT GCA TCT**

**181 GGA GTG GCG CTC CCA CTT ATG ATG CTG ATC TTT GGT GAC ATG ACA GAT AGC TTT GCA AGT**

**V G Q V S K Q S T Q M S E A D K R A M F**

**241 GTC GGT CAG GTG TCC AAG CAA TCT ACC CAG ATG TCT GAA GCT GAT AAG AGA GCT ATG TTT**

**241 GTA GGA AAC GTC TCT AAA AAC AGT ACT AAT ATG AGT GAG GCC GAT AAA AGA GCC ATG TTT**

**A K L E E E M T T Y A Y Y Y T G I G A G**

**301 GCA AAG TTA GAG GAG GAG ATG ACT ACT TAC GCA TAC TAC TAT ACC GGA ATT GGA GCC GGA**

**301 GCC AAA CTG GAG GAA GAA ATG ACC ACG TAC GCC TAC TAT TAC ACC GGG ATT GGT GCT GGT**

**V L I V A Y I Q V S F W C L A A G R Q I**

**361 GTT TTG ATT GTT GCA TAC ATC CAA GTT TCC TTC TGG TGC CTG GCC GCT GGA AGA CAA ATC**

**361 GTG CTC ATA GTT GCC TAC ATC CAG GTT TCA TTT TGG TGC CTG GCA GCT GGA AGA CAG ATA**

**H K I R Q K F F H A I M N Q E I G W F D**

**421 CAC AAG ATT AGA CAG AAG TTC TTC CAC GCT ATC ATG AAT CAA GAG ATT GGT TGG TTT GAC**

**421 CAC AAG ATC AGG CAG AAG TTT TTT CAT GCT ATA ATG AAT CAG GAG ATA GGC TGG TTT GAT**

**V H D V G E L N T R L T D D V S K I N E**

**481 GTG CAT GAC GTG GGA GAG CTC AAC ACC AGA CTT ACT GAC GAC GTG TCC AAG ATT AAC GAA**

**481 GTG CAT GAC GTT GGG GAG CTC AAC ACC CGG CTC ACA GAT GAT GTT TCC AAA ATT AAT GAA**

**G I G D K I G M F F Q A M A T F F G G F**

**541 GGA ATC GGT GAC AAG ATC GGA ATG TTC TTC CAG GCT ATG GCA ACT TTT TTC GGT GGT TTC**

**541 GGA ATT GGT GAC AAA ATC GGA ATG TTC TTC CAG GCA ATG GCA ACA TTT TTT GGT GGT TTT**

**I I G F T R G W K L T L V I L A I S P V**

**601 ATC ATC GGT TTC ACC CGC GGT TGG AAG TTG ACT CTT GTC ATT TTG GCC ATC TCT CCA GTC**

**601 ATA ATA GGA TTT ACC CGT GGC TGG AAG CTA ACC CTT GTG ATT TTG GCC ATC AGC CCT GTT**

**L G L S A G I W A K I L S S F T D K E L**

**661 TTG GGT CTT TCT GCT GGT ATT TGG GCC AAG ATT CTG TCT TCC TTC ACT GAC AAG GAA CTT**

**661 CTT GGA CTG TCA GCT GGT ATT TGG GCA AAG ATA TTG TCT TCA TTT ACT GAT AAG GAA CTC**

**H A Y A K A G A V A E E V L A A I R T V**

**721 CAT GCT TAC GCC AAA GCT GGA GCT GTT GCA GAA GAA GTC TTG GCT GCT ATC AGA ACC GTG**

**721 CAT GCT TAT GCA AAA GCT GGA GCA GTT GCT GAA GAA GTC TTA GCA GCC ATC AGA ACT GTG**

**I A F G G Q K K E L E R Y N N N L E E A**

**781 ATT GCT TTT GGT GGT CAG AAG AAG GAG CTT GAA AGA TAC AAC AAC AAC TTA GAA GAG GCT**

**781 ATT GCG TTT GGA GGA CAA AAG AAG GAA CTT GAA AGG TAC AAT AAC AAC TTG GAA GAA GCT**

**K R L G I K K A I T A N I S M G A A F L**

**841 AAG AGA TTG GGT ATC AAG AAG GCA ATT ACT GCT AAT ATC TCT ATG GGT GCT GCC TTT TTG**

**841 AAA AGG CTG GGG ATA AAG AAA GCT ATC ACG GCC AAC ATC TCC ATG GGT GCA GCT TTT CTC**

**L I Y A S Y A L A F W Y G T S L V I S K**

**901 CTG ATC TAC GCT TCA TAT GCT TTG GCT TTC TGG TAT GGT ACT TCC CTG GTC ATC TCT AAG**

**901 CTT ATC TAT GCA TCA TAT GCT CTG GCA TTC TGG TAT GGG ACT TCC TTG GTC ATC TCC AAA**

**E Y S I G Q V L T V F F S V L I G A F S**

**961 GAG TAC TCT ATC GGT CAG GTT TTG ACC GTC TTC TTC TCC GTC TTA ATT GGT GCT TTC TCT**

**961 GAA TAC TCT ATT GGA CAA GTG CTC ACT GTC TTC TTT TCC GTG TTA ATT GGA GCA TTC AGT**

**V G Q A S P N I E A F A N A R G A A Y E**

**1021 GTT GGA CAG GCT TCC CCT AAC ATT GAG GCC TTT GCT AAT GCC AGA GGT GCA GCT TAT GAG**

**1021 GTT GGA CAG GCA TCT CCA AAT ATT GAA GCC TTC GCC AAT GCA CGA GGA GCA GCT TAT GAA**

**V F K I I D N K P S I D S F S K S G H K**

**1081 GTC TTC AAG ATC ATC GAC AAC AAG CCA TCC ATT GAC TCT TTC TCC AAG TCC GGT CAC AAA**

**1081 GTC TTC AAA ATA ATT GAT AAT AAG CCC AGT ATA GAC AGC TTC TCA AAG AGT GGG CAC AAA**

**P D N I Q G N L E F K N I H F S Y P S R**

**1141 CCA GAC AAC ATT CAA GGA AAC CTG GAG TTC AAG AAC ATC CAC TTT TCT TAC CCT TCG CGA**

**1141 CCA GAC AAC ATA CAA GGA AAT CTG GAA TTT AAG AAT ATT CAC TTC AGT TAC CCA TCT CGA**

**K E V Q I L K G L N L K V K S G Q T V A**

**1201 AAA GAG GTC CAA ATC TTG AAG GGT TTG AAT CTG AAG GTC AAA TCT GGA CAA ACC GTT GCA**

**1201 AAA GAA GTT CAG ATC TTG AAG GGC CTC AAT CTG AAG GTG AAG AGC GGA CAG ACG GTG GCC**

**L V G N S G C G K S T T V Q L M Q R L Y**

**1261 TTG GTC GGA AAT TCT GGT TGT GGT AAG TCC ACC ACT GTT CAA CTT ATG CAG AGA TTA TAC**

**1261 CTG GTT GGC AAC AGT GGC TGT GGA AAA AGC ACA ACT GTC CAG CTG ATG CAA AGG CTC TAC**

**D P L D G M V S I D G Q D I R T I N V R**

**1321 GAC CCT TTG GAT GGA ATG GTC TCC ATT GAC GGA CAG GAC ATC CGT ACC ATT AAC GTC AGA**

**1321 GAC CCC CTA GAT GGC ATG GTC AGT ATC GAC GGA CAG GAC ATC AGA ACC ATC AAT GTG AGG**

**Y L R E I I G V V S Q E P V L F A T T I**

**1381 TAC CTG AGA GAG ATT ATC GGT GTT GTC TCC CAA GAG CCA GTC TTG TTT GCC ACT ACT ATC**

**1381 TAT CTG AGG GAG ATC ATT GGT GTG GTG AGT CAG GAA CCT GTG CTG TTT GCC ACC ACG ATC**

**A E N I R Y G R E D V T M D E I E K A V**

**1441 GCA GAG AAC ATC AGA TAT GGT CGT GAA GAC GTC ACC ATG GAC GAA ATT GAA AAG GCC GTT**

**1441 GCC GAG AAC ATT CGC TAT GGC CGA GAA GAT GTC ACC ATG GAT GAG ATT GAG AAA GCT GTC**

**K E A N A Y D F I M K L P H Q F D T L V**

**1501 AAG GAG GCT AAC GCT TAC GAT TTC ATC ATG AAG CTG CCA CAC CAA TTT GAC ACT TTA GTT**

**1501 AAG GAA GCC AAT GCC TAT GAC TTC ATC ATG AAA CTG CCC CAC CAA TTT GAC ACC CTG GTT**

**G E R G A Q L S G G Q K Q R I A I A R A**

**1561 GGT GAA CGT GGT GCT CAG TTG TCT GGT GGT CAA AAA CAA CGT ATC GCT ATT GCC CGG GCT**

**1561 GGT GAG AGA GGG GCG CAG CTG AGT GGG GGA CAG AAA CAG AGA ATC GCC ATT GCC CGG GCC**

**L V R N P K I L L L D E A T S A L D T E**

**1621 TTG GTC AGA AAC CCA AAG ATC TTG TTG TTA GAT GAG GCT ACC TCT GCT TTA GAC ACT GAG**

**1621 CTG GTC CGC AAT CCC AAG ATC CTT TTG TTG GAC GAG GCC ACC TCA GCC CTG GAT ACA GAA**

**S E A V V Q A A L D K A R E G R T T I V**

**1681 TCC GAG GCA GTT GTC CAA GCT GCC TTA GAC AAG GCC CGT GAG GGT AGA ACT ACC ATC GTT**

**1681 AGT GAA GCT GTG GTT CAG GCC GCA CTG GAT AAG GCT AGA GAA GGC CGG ACC ACC ATT GTG**

**I A H R L S T V R N A D V I A G F D G G**

**1741 ATT GCC CAC AGA TTG TCC ACT GTG AGA AAC GCT GAT GTT ATT GCA GGT TTT GAC GGT GGA**

**1741 ATA GCT CAT CGC TTG TCT ACC GTT CGT AAT GCT GAC GTC ATT GCT GGT TTT GAT GGT GGT**

**V I V E Q G N H D E L M R E K G I Y F K**

**1801 GTT ATT GTC GAA CAA GGA AAC CAC GAC GAG CTC ATG AGA GAG AAG GGA ATC TAC TTC AAG**

**1801 GTC ATT GTG GAG CAA GGA AAT CAT GAT GAG CTC ATG AGA GAA AAG GGC ATT TAC TTC AAA**

**L V M T Q T A G N E I E L G N E A C K S**

**1861 TTG GTG ATG ACT CAA ACC GCT GGA AAC GAG ATT GAA CTT GGA AAT GAG GCT TGC AAG TCT**

**1861 CTT GTC ATG ACA CAG ACA GCA GGA AAT GAA ATT GAA TTA GGA AAT GAA GCT TGT AAA TCT**

**K D E I D N L D M S S K D S G S S L I R**

**1921 AAG GAC GAG ATT GAT AAC CTT GAC ATG TCT TCC AAG GAC TCT GGT TCC TCT CTG ATT AGA**

**1921 AAG GAT GAA ATT GAT AAT TTA GAC ATG TCT TCA AAA GAT TCA GGA TCC AGT CTA ATA AGA**

**R R S T R K S I C G P H D Q D R K L S T**

**1981 AGA CGT TCC ACT AGA AAG TCC ATT TGT GGT CCA CAC GAT CAA GAC AGA AAG TTG TCC ACC**

**1981 AGA AGA TCA ACT CGC AAA AGC ATC TGT GGA CCA CAT GAC CAA GAC AGG AAG CTT AGT ACC**

**K E A L D E D V P P A S F W R I L K L N**

**2041 AAA GAA GCT TTG GAC GAA GAT GTC CCA CCT GCC TCT TTC TGG CGG ATC CTT AAA TTG AAT**

**2041 AAA GAG GCC CTG GAT GAA GAT GTA CCT CCA GCT TCC TTT TGG CGG ATC CTG AAG TTG AAT**

**S T E W P Y F V V G I F C A I I N G G L**

**2101 TCA ACT GAG TGG CCT TAC TTC GTG GTT GGT ATC TTC TGT GCC ATC ATT AAC GGT GGT TTG**

**2101 TCA ACT GAA TGG CCT TAT TTT GTG GTT GGT ATA TTC TGT GCC ATA ATA AAT GGA GGC TTA**

**Q P A F S V I F S K V V G V F T N G G P**

**2161 CAG CCA GCT TTT TCT GTG ATC TTC TCC AAG GTT GTG GGT GTT TTT ACT AAT GGT GGT CCA**

**2161 CAG CCA GCA TTC TCC GTA ATA TTT TCA AAA GTT GTA GGG GTT TTT ACA AAT GGT GGC CCC**

**P E T Q R Q N S N L F S L L F L I L G I**

**2221 CCA GAA ACC CAA AGA CAG AAC TCT AAC TTG TTC TCC CTG TTG TTC TTG ATC CTA GGT ATC**

**2221 CCT GAA ACC CAG CGG CAG AAC AGC AAC TTG TTT TCC TTG TTG TTT CTG ATC CTT GGG ATC**

**I S F I T F F L Q G F T F G K A G E I L**

**2281 ATC TCT TTC ATT ACT TTC TTC CTG CAA GGT TTC ACT TTT GGT AAA GCC GGT GAG ATT CTG**

**2281 ATT TCT TTC ATT ACA TTT TTT CTT CAG GGC TTC ACA TTT GGC AAA GCT GGA GAG ATC CTC**

**T K R L R Y M V F K S M L R Q D V S W F**

**2341 ACC AAG AGA TTG AGA TAC ATG GTC TTC AAG TCT ATG TTG CGT CAA GAT GTC TCT TGG TTC**

**2341 ACC AAG CGA CTC CGA TAC ATG GTT TTC AAA TCC ATG CTG AGA CAG GAT GTG AGC TGG TTT**

**D D P K N T T G A L T T R L A N D A A Q**

**2401 GAT GAT CCT AAG AAC ACT ACT GGT GCC TTG ACT ACC AGA TTG GCT AAT GAC GCT GCC CAA**

**2401 GAT GAC CCT AAA AAC ACC ACC GGA GCA CTG ACC ACC AGG CTC GCC AAC GAT GCT GCT CAA**

**V K G A T G S R L A V I F Q N I A N L G**

**2461 GTT AAA GGA GCT ACT GGT AGT CGA CTT GCT GTT ATT TTC CAG AAC ATT GCC AAC TTG GGA**

**2461 GTG AAA GGG GCT ACA GGG AGT CGA CTT GCT GTG ATT TTC CAG AAC ATA GCA AAT CTT GGG**

**T G I I I S L I Y G W Q L T L L L L A I**

**2521 ACC GGA ATT ATC ATC TCC TTG ATC TAC GGT TGG CAG CTT ACT TTG TTA CTG TTG GCT ATC**

**2521 ACA GGA ATC ATC ATA TCC CTA ATC TAT GGC TGG CAA CTA ACA CTT TTA CTC TTA GCA ATT**

**V P I I A I A G V V E M K M L S G Q A L**

**2581 GTC CCT ATC ATT GCC ATT GCC GGT GTT GTC GAG ATG AAG ATG CTG TCT GGT CAA GCC TTG**

**2581 GTA CCC ATC ATT GCG ATA GCA GGA GTG GTT GAA ATG AAA ATG TTG TCT GGA CAA GCA CTG**

**K D K K E L E G S G K I A T E A I E N F**

**2641 AAA GAC AAG AAG GAG TTG GAG GGT TCC GGA AAG ATT GCT ACC GAA GCA ATT GAG AAC TTC**

**2641 AAA GAT AAG AAG GAA CTA GAA GGT TCT GGA AAG ATT GCT ACG GAA GCA ATT GAA AAC TTC**

**R T V V S L T R E Q K F E T M Y A Q S L**

**2701 CGT ACT GTT GTT TCT CTT ACT CGT GAG CAG AAG TTC GAG ACT ATG TAC GCT CAA TCC TTA**

**2701 CGC ACT GTT GTC TCT TTG ACT CGC GAG CAG AAG TTT GAA ACC ATG TAT GCC CAG AGC TTG**

**Q I P Y R N A M K K A H V F G I T F S F**

**2761 CAG ATT CCA TAC CGT AAC GCT ATG AAA AAG GCT CAC GTT TTC GGT ATT ACC TTC TCT TTC**

**2761 CAG ATA CCA TAC AGA AAT GCG ATG AAG AAA GCA CAC GTG TTT GGG ATC ACG TTC TCC TTC**

**T Q A M M Y F S Y A A C F R F G A Y L V**

**2821 ACC CAA GCT ATG ATG TAC TTC TCC TAC GCT GCC TGT TTC AGA TTC GGT GCT TAT TTG GTG**

**2821 ACC CAG GCC ATG ATG TAT TTT TCT TAT GCT GCT TGT TTC CGG TTC GGT GCC TAC TTG GTG**

**T Q Q L M T F E N V L L V F S A I V F G**

**2881 ACT CAG CAA TTG ATG ACC TTC GAG AAC GTT CTA CTA GTA TTC TCT GCT ATT GTT TTC GGA**

**2881 ACA CAA CAA CTC ATG ACT TTT GAA AAT GTT CTA CTA GTA TTC TCA GCT ATT GTC TTT GGT**

**A M A V G Q V S S F A P D Y A K A T V S**

**2941 GCC ATG GCA GTT GGT CAA GTT TCC TCT TTC GCC CCT GAT TAT GCC AAG GCT ACT GTC TCT**

**2941 GCC ATG GCA GTG GGG CAG GTC AGT TCA TTC GCT CCT GAC TAT GCG AAA GCC ACA GTG TCA**

**A S H I I R I I E K T P E I D S Y S T Q**

**3001 GCA TCC CAC ATC ATT AGA ATC ATT GAG AAG ACC CCA GAG ATT GAT TCT TAC TCC ACC CAA**

**3001 GCA TCC CAC ATC ATC AGG ATC ATT GAG AAA ACC CCC GAG ATT GAC AGC TAC AGC ACG CAA**

**G L K P N M L E G N V Q F S G V V F N Y**

**3061 GGT TTG AAG CCT AAT ATG CTG GAG GGT AAC GTT CAG TTC TCT GGT GTT GTC TTC AAT TAC**

**3061 GGC CTA AAG CCG AAT ATG TTG GAA GGA AAT GTG CAA TTT AGT GGA GTC GTG TTC AAC TAT**

**P T R P S I P V L Q G L S L E V K K G Q**

**3121 CCA ACT AGA CCA TCT ATT CCA GTT CTG CAA GGT CTT TCT TTG GAG GTT AAG AAG GGT CAA**

**3121 CCC ACC CGA CCC AGC ATC CCA GTG CTT CAG GGG CTG AGC CTT GAG GTG AAG AAG GGC CAG**

**T L A L V G S S G C G K S T V V Q L L E**

**3181 ACC CTT GCC TTG GTT GGT TCT TCC GGT TGT GGT AAG TCC ACT GTT GTT CAG CTT TTG GAG**

**3181 ACG CTG GCC CTG GTG GGC AGC AGT GGC TGC GGG AAG AGC ACA GTG GTC CAG CTG CTC GAG**

**R F Y D P M A G S V F L D G K E I K Q L**

**3241 CGT TTC TAC GAT CCA ATG GCC GGT TCT GTG TTT CTA GAT GGA AAG GAG ATC AAG CAG TTG**

**3241 CGC TTC TAC GAC CCC ATG GCT GGA TCA GTG TTT CTA GAT GGC AAA GAA ATA AAG CAA CTG**

**N V Q W L R A Q L G I V S Q E P I L F D**

**3301 AAC GTC CAA TGG TTG AGA GCC CAA TTG GGT ATT GTG TCT CAA GAG CCT ATT CTT TTC GAC**

**3301 AAT GTC CAG TGG CTC CGA GCA CAG CTG GGC ATT GTG TCC CAA GAG CCC ATT CTC TTT GAC**

**C S I A E N I A Y G D N S R V V S Y E E**

**3361 TGC AGC ATT GCA GAG AAC ATT GCT TAT GGT GAC AAC TCC AGA GTT GTG TCC TAT GAG GAA**

**3361 TGC AGC ATC GCA GAG AAC ATT GCC TAC GGA GAC AAC AGC CGG GTC GTG TCT TAT GAG GAG**

**I V R A A K E A N I H Q F I D S L P D K**

**3421 ATC GTT AGA GCT GCC AAA GAG GCT AAC ATT CAC CAG TTC ATC GAC TCT CTT CCT GAT AAG**

**3421 ATT GTG AGG GCA GCC AAG GAG GCC AAC ATC CAC CAG TTC ATC GAC TCG CTA CCT GAT AAA**

**Y N T R V G D K G T Q L S G G Q K Q R I**

**3481 TAC AAC ACC AGA GTC GGA GAC AAA GGT ACT CAA TTG TCT GGT GGT CAA AAG CAA AGA ATC**

**3481 TAC AAC ACC AGA GTA GGA GAC AAA GGC ACT CAG CTG TCG GGT GGG CAG AAG CAG CGC ATC**

**A I A R A L V R Q P H I L L L D E A T S**

**3541 GCT ATT GCT CGT GCT CTG GTT AGA CAA CCT CAC ATC TTG CTG TTG GAC GAA GCT ACC TCT**

**3541 GCC ATC GCA CGC GCC CTC GTC AGA CAG CCT CAC ATT TTA CTT CTG GAC GAA GCA ACA TCA**

**A L D T E S E K V V Q E A L D K A R E G**

**3601 GCT TTG GAC ACT GAA TCT GAG AAA GTC GTT CAA GAG GCT CTG GAT AAG GCC AGA GAA GGA**

**3601 GCT CTG GAT ACA GAA AGT GAA AAG GTT GTC CAG GAA GCG CTG GAC AAA GCC AGG GAA GGC**

**R T C I V I A H R L S T I Q N A D L I V**

**3661 AGA ACT TGT ATC GTC ATT GCT CAC CGT TTG TCC ACT ATT CAA AAC GCT GAC CTG ATT GTG**

**3661 CGC ACC TGC ATT GTG ATC GCT CAC CGC CTG TCC ACC ATC CAG AAC GCG GAC TTG ATC GTG**

**V I Q N G K V K E H G T H Q Q L L A Q K**

**3721 GTC ATT CAA AAC GGT AAG GTC AAG GAG CAT GGA ACT CAT CAA CAG CTG TTG GCT CAG AAA**

**3721 GTG ATT CAG AAC GGC AAG GTC AAG GAG CAC GGC ACC CAC CAG CAG CTG CTG GCG CAG AAG**

**G I Y F S M V S V Q A G A K R S L E H H**

**3781 GGT ATC TAC TTC TCT ATG GTT TCC GTC CAA GCT GGA GCC AAA AGA TCT CTC GAG CAT CAT**

**3781 GGC ATC TAC TTC TCA ATG GTC AGT GTG CAG GCT GGA GCA AAG CGC TCA TAC GTA CAT CAC**

**H H H H ***

**3841 CAT CAT CAT CAT TGA**

**3841 CAT CAC CAT CAC TGA**

The changes in the nucleotide sequence of *Opti-mdr3* compared to wild-type *mdr3* are marked in **red**.
